# Orientation pinwheels in primary visual cortex of a highly visual marsupial

**DOI:** 10.1126/sciadv.abn0954

**Published:** 2022-09-30

**Authors:** Young Jun Jung, Ali Almasi, Shi H. Sun, Molis Yunzab, Shaun L. Cloherty, Sebastien H. Bauquier, Marilyn Renfree, Hamish Meffin, Michael R. Ibbotson

**Affiliations:** ^1^ National Vision Research Institute, Melbourne, VIC, Australia.; ^2^ Optalert Limited, Melbourne, VIC, Australia.; ^3^ Department of Neurosurgery, Massachusetts General Hospital, Harvard Medical School, Boston, MA 02114, USA.; ^4^ School of Engineering, RMIT University, Melbourne, VIC, Australia.; ^5^ Veterinary Hospital, Faculty of Veterinary and Agricultural Sciences, The University of Melbourne, Melbourne, VIC, Australia.; ^6^ School of BioSciences, The University of Melbourne, Melbourne, VIC, Australia.; ^7^ Department of Biomedical Engineering, The University of Melbourne, Melbourne, VIC, Australia.; ^8^ Department of Optometry and Vision Sciences, The University of Melbourne, Melbourne, VIC, Australia.

## Abstract

Primary visual cortices in many mammalian species exhibit modular and periodic orientation preference maps arranged in pinwheel-like layouts. The role of inherited traits as opposed to environmental influences in determining this organization remains unclear. Here, we characterize the cortical organization of an Australian marsupial, revealing pinwheel organization resembling that of eutherian carnivores and primates but distinctly different from the simpler salt-and-pepper arrangement of eutherian rodents and rabbits. The divergence of marsupials from eutherians 160 million years ago and the later emergence of rodents and rabbits suggest that the salt-and-pepper structure is not the primitive ancestral form. Rather, the genetic code that enables complex pinwheel formation is likely widespread, perhaps extending back to the common therian ancestors of modern mammals.

## Teaser

Pinwheel orientation preference maps, similar to those in cats and primates, are reported in marsupial primary visual cortex.

## INTRODUCTION

In mammals, the spatial relationships within an image are preserved in the retina, and, in turn, this retinotopic arrangement continues in the visual field maps of the primary visual cortex (V1) (*1*–*3*). In cats, ferrets, tree shrews, and primates (*4*–*8*), which are all eutherian mammals, neurons viewing each patch of visual space are further segregated into pinwheel-like structures in which each pinwheel segment codes specific edge orientations. Thus, a full pinwheel codes all orientations in one patch of space in an ordered radial pattern (*4*–*8*). These modular maps are thought to exist to minimize neuronal wiring length and maximize coverage for efficient information processing (*9*, *10*). Rodents and rabbits, which are also eutherian mammals, have the same retinotopic representation, but orientation-selective neurons within a given visual field location are distributed in a nonperiodic fashion, generating salt-and-pepper orientation preference (OP) maps (*11*, *12*). Some studies (*13*–*15*) report spatial clustering at very short distances in mouse and agouti, where neighboring neurons exhibit similar OPs, but the neurons are not organized into pinwheel orientation maps. This is the case even in the squirrel (*12*), which is a highly visual rodent with a V1 similar in all obvious respects to that of the eutherian ferret, which has a pinwheel orientation map (*5*). Despite both species having complex visual behaviors and similar cortices, orientation-selective cells are arranged in very different ways, suggesting that there are at least two ways that the cortex can be organized. Other factors also differ between ferrets and squirrels, notably their different retinal structures, which have been matched through evolution to their visual habitats (*16*). Unlike in rodents, the ferret has higher central (C) compared to peripheral (P) cell density in the retina (i.e., a high CP ratio), which has been shown to correlate strongly with the existence of pinwheel maps in a range of species (*17*), leaving open the possibility of retinal design being an important factor in influencing cortical design.

**Fig. 1.**
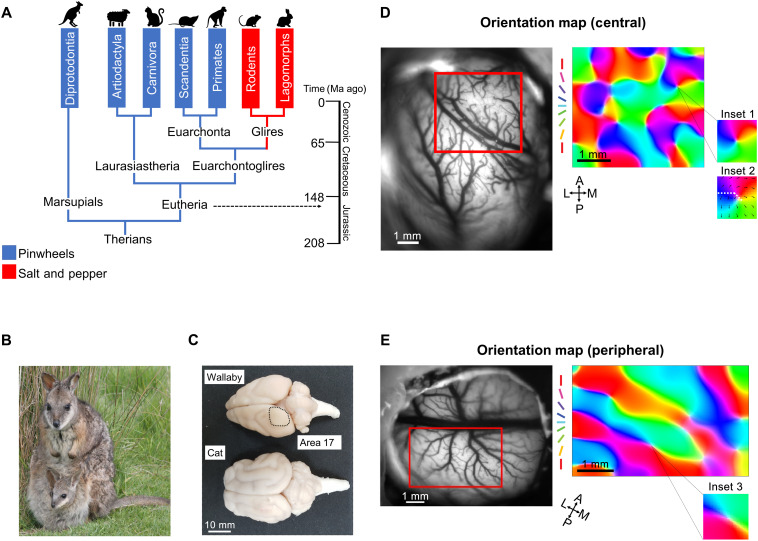
Orientation maps of wallaby visual cortex. (**A**) A simplified mammalian phylogenetic tree. Mammalian order names with blue backgrounds have a pinwheel-like organization of orientation selectivity in V1. Order names with backgrounds in red have salt-and-pepper cortical organization. Ma ago, million years ago. (**B**) A standing tammar wallaby with pouch young. Photo credit: M. Renfree, The University of Melbourne. (**C**) Size comparison of a wallaby and a cat brain. Photo credit: Y. J. Jung, National Vision Research Institute. The vasculature images of the entire cortical area and the OP maps obtained in the selected region of interest (in red) from the (**D**) central and (**E**) peripheral visual field of wallaby V1. Preferred orientations are color-coded according to the scheme in the legend. Inset 1 shows a single pinwheel from the central map. Inset 2 shows preferred direction using black arrows overlayed on the OP map from inset 1. Direction preference varies smoothly with orientation except at the fracture (white dashed line) where contiguous cortical regions have opposite preferences. Inset 3 shows a single pinwheel from the peripheral map. A, anterior; P, posterior; M, medial; L, lateral.

Recent studies have found that the orientation column layout follows a common architecture in disparate mammalian lineages (i.e., tree shrews, ferrets, galago, and mouse lemurs) (*9*, *18*, *19*), which implies that there is a universal constraining developmental mechanism involved in generating orientation columns. Kaschube *et al*. (*9*) suggest that the common orientation column layout is a natural consequence of self-organizing cortical networks dominated by long-range interactions in the brain. Hence, it has been suggested that self-organizing dynamical systems are stronger drivers than genetic prespecification or environmental selective pressures for the development of orientation columns. The common columnar organization is size invariant; even the smallest primate, the mouse lemur with a small V1 (48 mm^2^), has the same columnar organization seen in far larger primates (e.g., macaque V1, 1181 mm^2^) (*18*). There is an ongoing debate regarding whether this organization developed independently in mammals of the clades Laurasiatheria and Euarchonta or whether this architecture is inherited from a common ancestor but lost in rodents and lagomorphs (Fig. 1A) (*18*–*21*).

An obvious approach to understanding the origins of cortical map architecture would be to measure maps in many species from all groups of mammals—a very challenging task. Instead, we decided to study a highly visual marsupial, the wallaby (Fig. 1B), which sits on a phylogenetic lineage that originated 160 million years ago at the division point between eutherians and marsupials (Fig. 1A) (*22*). Although some brain structures differ between these groups, such as the lack of a marsupial corpus callosum (*23*), the general identities of visual brain areas and brain sizes between species with similar body mass (e.g., cat and wallaby) are similar (Fig. 1C). However, we do not yet have any information about the organization of feature selectivity in marsupial V1. If highly visual marsupials have salt-and-pepper organization in V1, then cats and primates might have evolved pinwheels relatively recently. This would be regarded as a newly developed apomorphic trait. Conversely, if marsupials have pinwheels, then it is likely that the salt-and-pepper organization in rodents and rabbits is the apomorphic trait (red branches; Fig. 1A). In this case, it is also likely that the ancestral therian mammals had the capacity for pinwheel structures in V1.

## RESULTS

We used optical imaging of intrinsic signals (*24*) and single-cell recordings with multichannel electrode arrays to determine the organization of feature-selective neurons in wallaby V1. Wallabies were bred for use in research and lived in open fields, providing natural visual environments in which to develop. The visual field representation and the anatomical structure of V1 have been comprehensively established in the wallaby (*25*, *26*), which is a small (4- to 8-kg) kangaroo. Eye divergence is approximately 45°, but retinal ganglion cells (RGCs) that provide input to the cortical pathway are overrepresented in the lateral region of the retina associated with forward viewing (*25*).

Optical imaging of wallaby V1 during the presentation of moving grating stimuli revealed clear orientation pinwheels (Fig. 1D). The stimulus screen (60° wide and 30° high) was placed in two positions, directly in front to stimulate the region of binocular overlap and laterally to stimulate the monocular peripheral visual field. We show orientation columns observed in the central and peripheral field representations, respectively (monocular stimulation of the contralateral eye) (Fig. 1, D and E). For central vision, the orientation columns are arranged radially around pinwheel centers (Fig. 1D). Pinwheels are also present in the peripheral representation, but they are embedded in a more banded structure (Fig. 1E).

Using the statistical method developed by Kaschube *et al*. (*9*), we calculated the pinwheel density (ρ) as the mean number of pinwheels per orientation-hypercolumn area (Λ^2^). The area of an orientation hypercolumn was calculated as the square of the local column spacing (Λ), which is defined as the interval between areas responding to the same orientation (see Materials and Methods). This method of calculating the pinwheel density characterizes the spatial arrangement of the orientation columns independently of their absolute size. The average pinwheel density in the central visual field for two animals was 2.81 [2.97 and 2.65], with an average Λ of 1.42 mm [1.55 and 1.28 mm]. Figure 1E shows the arrangement of OPs in the peripheral visual field representation. For this region of the visual field, orientation-selective cells are arranged in bands rather than columns. Pinwheel centers could still be observed at the edges of these bands, but the segments of the pinwheels were elongated (Fig. 1E). The average pinwheel density (*n* = 4) was lower, at 2.53 [2.37, 1.95, 2.95, and 2.86], with an average Λ of 1.44 mm [1.74, 1.14, 1.27, and 1.61 mm]. Unlike the geometric lattice–like organization observed in carnivores and primates (*4*–*8*), the pinwheels were more uneven and sparse in wallaby V1. We found that the orientation maps in wallaby V1 composed of many banded regions, especially in the periphery where a large portion of cortical space represented cardinal angles. On average, 65% of the map area represented cardinal angles centrally (*n* = 2) and 76% peripherally (*n* = 4). Figure S6 shows OP maps from all six imaged animals, including the proportion of cortical area representing different orientations.

When generating OP maps, we used drifting gratings, i.e., gratings were moved back and forth perpendicular to the presented orientation. For the OP maps, we combined the responses for both motion directions. We also used the directional data independently to create maps of direction selectivity (Fig. 1D, inset 2). As in other investigated species (*4*, *27*), we found that direction preferences were coded in discrete regions of cortex. The direction preferences not only changed gradually in a radial pattern from one area to the next but also contained “fractures” where opposite directions were coded on different sides of a fracture line.

**Fig. 2.**
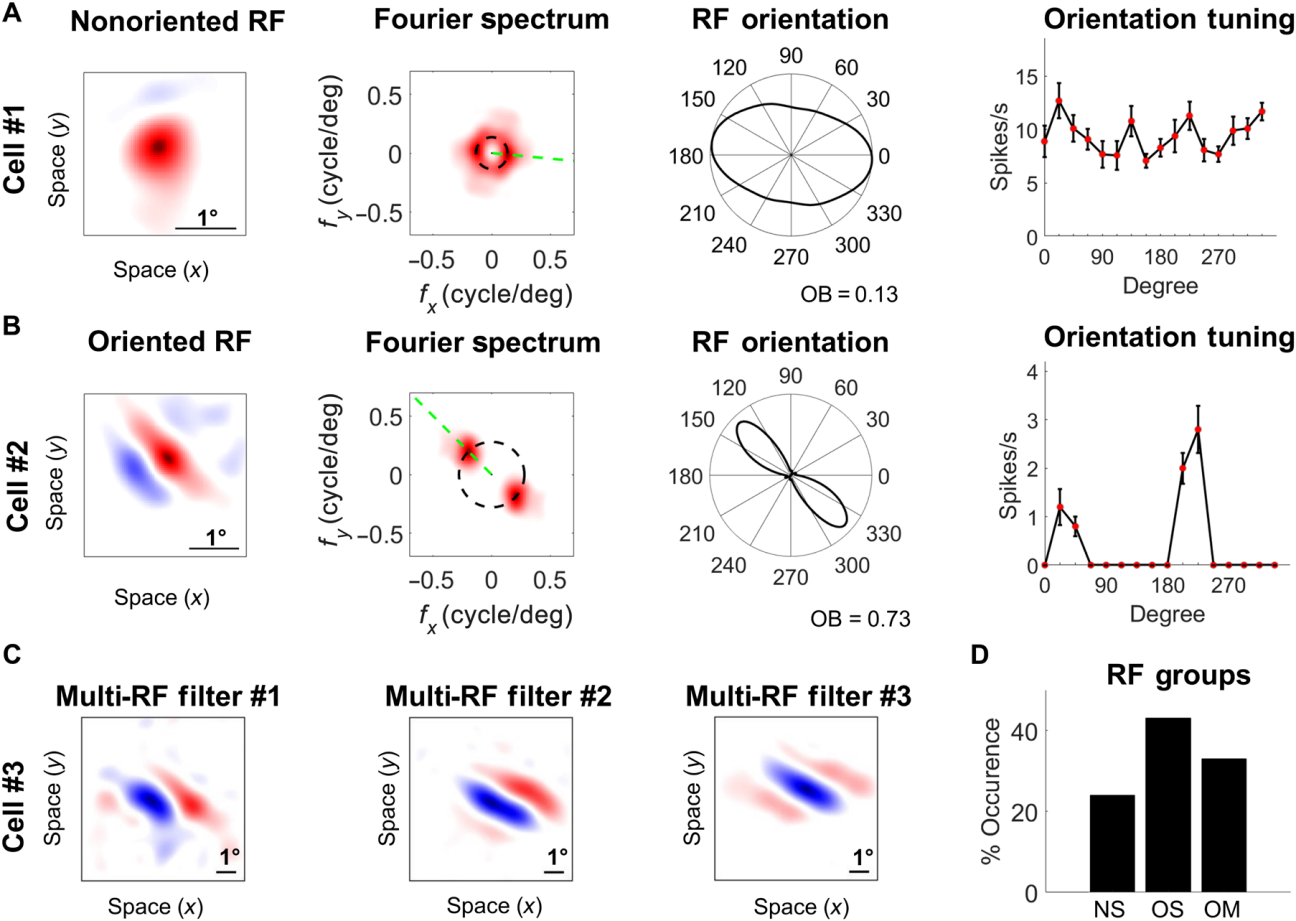
Electrophysiology recordings of wallaby V1 cells. (**A** and **B**) Two example single units with single nonoriented (A) and oriented RF filters (B). Red and blue represents ON and OFF responses, respectively. Black scale bar indicates 1° of visual field. The two-dimensional Fourier amplitude spectrum of the RF filters is shown (second from left), with the *x* and *y* axes indicating the spatial frequency in cycles per degree. The intensity of the red color indicates the amplitude of the Fourier spectrum. The black dashed circle indicates the preferred spatial frequency of the filter. The green dashed line indicates the preferred orientation of the filter. The graphs third from the left show tuning polar plots obtained by sampling the amplitude spectrum at the preferred spatial frequency. Orientation selectivity of cells is quantified with an orientation bias (OB) index, which ranges from 0 (no selectivity) to 1 (narrow selectivity). The right-hand plots show orientation tuning curves obtained when stimulating the cells with drifting sinusoidal gratings in 16 directions equally spaced between 0° and 360°. (i.e., 0° is vertical bar drifting right). Hence, we see a 90° difference between the RF polar plot and orientation tuning curve (**C**) Example of the spatial structures of the three filters that make up the RF of a multifilter cell. Note that the three filters have similar preferred orientations, but the ON and OFF bands are displaced relative to each other. (**D**) Bar graph showing the occurrences of nonoriented single-filter (NS) units, oriented single-filter (OS) units, and oriented multifilter (OM) units in the population (*n* = 195).

The fact that these robust OP maps could be measured using intrinsic signal imaging suggests that wallaby V1 is dominated by orientation-selective neurons. Early recordings from V1 in two other marsupials, the American opossum (*28*, *29*) and Australian possum (*30*), reported low percentages of orientation-selective cells (<50%), which contrasted with the high proportions (70 to 90%) reported in eutherian V1 (*31*, *32*). An interpretation of previous findings might have been that orientation selectivity had less importance in marsupials or only fully emerged later in the cortical pathway. However, in the present study, we find that around 70% of neurons in wallaby V1 were orientation selective (Fig. 2D), suggesting that the reported percentages might have been technique dependent or that there are real differences in percentages between marsupial species. In earlier studies (*26*, *28*, *30*), cortical neurons were recorded one at a time using stimuli such as bars and gratings, which may bias the sampling. Therefore, here, we used multichannel electrode arrays combined with white noise stimuli and applied recently developed objective receptive field (RF) analysis techniques (*33*) to quantify orientation selectivity and correlate single-cell tuning properties with the OP maps obtained from optical imaging (Fig. 2). Our stimuli were always presented monocularly, with an eye patch to prevent stimulation of the fellow eye.

**Fig. 3.**
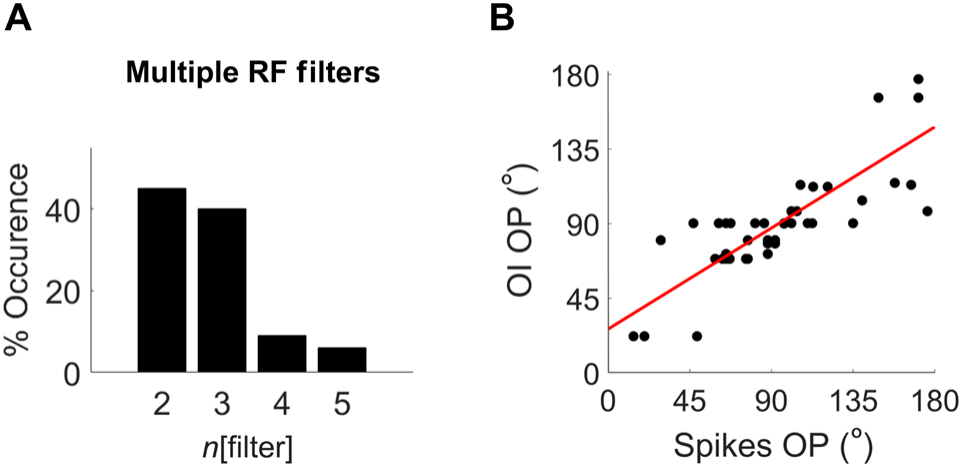
Electrophysiology recordings of wallaby V1 cells. (**A**) Bar graph showing the occurrences of cells with two to five filters. (**B**) Plot of OP measured from the single-unit recordings and the corresponding OP maps obtained through optical imaging (OI). Note that only single-unit recordings from superficial layers of the cortex (i.e., channels between 200 and 1000 μm) were used in this analysis. The red line is the least squares fit (*n* = 49, *r*^2^ = 0.676, *P* = 0.001) with a median absolute difference of 8°.

We recorded from 195 well-isolated single units (Fig. 2 and fig. S4). Using a nonlinear input model of RFs, it is possible to extract the structure of the spatial filters that created each unit’s feature selectivity (*33*). A spatial filter in this context describes the spatial features in the image that generate responses from a given cell. Where cells have more than one filter, their filter selectivity may be combined. We estimated one or more spatial filters for all recorded neurons (Fig. 2) and quantified the orientation selectivity of every filter using an orientation bias (OB) index (see Materials and Methods). We then assigned each recorded neuron to one of three possible categories: nonoriented RFs with a single filter (24%), oriented RFs with a single filter (43%), and oriented RFs with multiple filters (33%) (Fig. 2D). The same analysis of neurons in cat visual cortex (*n* = 325 cells) revealed comparable proportions of 29, 47, and 24%, respectively (*33*), suggesting little obvious difference at the population level in the basic properties of RFs in wallaby and cat. The nonoriented RFs in wallaby were sensitive to changes in luminance within their RFs, either preferring luminance increments (ON) or decrements (OFF). When the nonoriented RFs were tested with moving grating stimuli, they exhibited little directional bias (Fig. 2A, cell #1). The oriented RFs with a single subunit always had multiple, elongated bands that were selective to ON or OFF contrast (Fig. 2B, cell #2). Of the neurons with multiple filters (Fig. 2C, cell #3), 45% had two filters (Fig. 3A), and 55% had >2 filters. In most cases, the filters were similar to those found in the single-filter cells. Using the same techniques, in cat cortex, around 80% of multifilter RFs had only two filters (*33*), which shows that more cells in wallaby cortex have the capacity for multifeature selectivity than in cats. We speculate that in cat, processing may be spread over multiple cortical areas (e.g., areas 17, 18, 19, and 21a) (*34*), while in wallaby, more sophisticated processing is compressed within V1 (i.e., area 17). Considerable variation in the strata at which various types of processing occur has been noted across mammals (*35*). The RFs of neurons had similar orientation and filter characteristics in the central and peripheral representations, but the RFs (measured along their longest axes) were significantly smaller in the former (2.3° ± 0.96, means ± SD) compared to the latter (3.1° ± 0.97, means ± SD) (fig. S4).

The OPs of single units recorded in the superficial layers (*n* = 49) correlated strongly with the OP maps (*r*^2^ = 0.676, *P* = 0.001; Fig. 3B), with a median absolute difference of 8° (see Fig. 3B and fig. S5). To assess whether wallaby cortex had orientation-selective columns throughout its depth, we calculated the SDs of OPs recorded along each vertically oriented recording track to determine the modularity in the vertical axis. The average SD in OP between cells was 23.84° ± 10.85 (means ± SD, *n* = 16 tracks), which indicates a small variation in OPs in a given track, supporting the existence of columns.

## DISCUSSION

### Importance of genetics

Here, we show that a marsupial, the tammar wallaby, has a pinwheel OP map in V1, which appears quite similar to the map observed in cat cortex. It is now possible to see that pinwheel cortical structures are present in the majority of mammalian lines, including both the main infraclasses, marsupials and eutherians (Fig. 1A). What now stands out very clearly is that species in the relatively recently evolved clade Glires (rodents and rabbits) appear to be the exception to the rule. Many species belonging to the Glires have been studied, including mice, rats, squirrels, agouti, and rabbits (*11*, *15*, *35*). Despite living in widely different environments and displaying a wide range of behaviors, all members of the clade Glires have salt-and-pepper OP maps. This is true even for the highly visual rodent, the agouti (*13*), which has a highly differentiated V1 with a surface area similar to the nonrodent cat (*4*), the latter having pinwheel OP maps. Are species within the clade Glires genetically predetermined to have a salt-and-pepper map? This is possible, but environmental and behavioral differences with other clades cannot be ignored (see the discussion of retinal design below). Another clade that has been studied extensively is the Euarchonta, which includes the primates (*8*, *9*, *18*) and tree shrews (Scandentia) (*6*). All of the cortices from the measured species belonging to the Euarchonta have pinwheel designs. It might be concluded that members of the clade Euarchonta are predetermined to have pinwheel maps, but it is also evident that primates tend to have quite complex visual behaviors, leaving open the possibility that behavior and environment are major drivers toward having pinwheel maps. Only a few scattered species that are not rodents or primates have been examined, notably the cat (*4*) and ferret (both from the order Carnivora) (*36*) and sheep (order Artiodactyla) (*37*), all of which have been shown to have pinwheel-type maps.

Fundamentally, it would be useful to know which came first; i.e., is the salt-and-pepper or the pinwheel structure the ancestral form? Schmidt and Wolf (*19*) suggest that the salt-and-pepper map may be the ancestral form and “that convergent evolution of the pinwheel phenotype may have arisen as an adaptation to common visual environments in multiple mammalian clades.” They further state “that new primate genes might have led to neocortical expansion, which, in turn, may have promoted high visual acuity (*38*–*39*), along with other higher functions in primates.” If we accept that the salt-and-pepper design is the ancestral form, then the finding that a highly visual marsupial also has pinwheels would suggest that similar genetic developments might have led to neocortical expansion in that species too through convergent evolution. The general evolutionary pressure toward increased brain size in a particular species might encourage innate developmental mechanisms toward a pinwheel OP structure because this offers advantages in processing efficiency (*11*). However, the Agouti has the highest level of neocorticalization in any rodent, along with a cortex with a large surface area, and it still has a salt-and-pepper OP map in cortex (*19*).

Taking an alternate view, it is possible based on the limited existing data that pinwheels might be the ancestral design because they are present in all mammalian branches studied so far, except the Glires (Fig. 1A). If pinwheels are the ancestral form, then it is possible that, in the Glires, selected genes for controlling the regulation of long-range horizontal axons were switched off or lost (*39*) during the division of the Euarchontoglires clade. These long-range horizontal axons target both excitatory and inhibitory local neuron populations. Kaschube *et al*. (*9*) suggest that controlling the overall synaptic input balance is important for the generation of a pinwheel design. Pinwheels being the ancestral form are the opposite argument to the notion that highly visual species (e.g., primates, cats, ferrets, sheep, and wallabies) each independently created new genes. If this theory is right, then, in the Glires, the genes that encode the protein machinery necessary for dynamic, activity-dependent plasticity and the elaboration of long-range connections across columns may have become selectively neutral (*23*, *32*).

Given that rodents have salt-and-pepper maps, it is likely that studies using rodents alone cannot offer all of the required information regarding the genetics of pinwheel formation. A way of moving toward identifying genes responsible for pinwheel formation is to find species that straddle the border between salt-and-pepper and pinwheel structures. Species with salt-and-pepper designs may have switched off or lost particular genes, or, alternatively, species with pinwheels may have all evolved or activated genes that lead to pinwheel designs. It is also the case that the growing evidence for microcolumns in rodents (*13*–*15*) might reveal further genetic complexity underlying cortical map formation.

### Importance of environment

There is a good precedent for the influence of the environment on the evolution of visual systems. Different mammalian species have unique regions of higher cell density in their retinas, which are directly guided by selective ecological and developmental pressures (*16*, *40*). For example, frontal-eyed species have centralized retinal regions of high cell density (e.g., primates, cats, tree kangaroos, and megabats) (*41*). These animals often live in arboreal environments or are predators, both with a need for high-resolution, binocular vision. In contrast, lateral-eyed animals often have extended horizontal bands of moderate retinal cell density that span the entire horizon (e.g., rabbits, sheep, plains and kangaroos) (*42*–*44*). The argument goes that, in these species, finding food is as easy as bending down to eat the grass below them but detecting predators is the overriding environmental need, thus requiring panoramic fields of view. However, even prey animals such as sheep have the ability to recognize complex objects of interest, such as human faces (*45*). As a result, some animals with a visual streak also have a patch of high-resolution retina within that part of the retina that looks directly forward, which may be displaced laterally in the retina. The third type of retinal design is usually found in small animals that have their heads very close to the ground, e.g., mice (*46*). The mouse has a uniform retinal cell density and no OP maps in cortex. In dogs that have been selectively bred to have short noses (e.g., pugs), the retina has an area centralis (*47*), and dogs that have been bred to have long noses and lateral eyes (e.g., greyhounds) have a visual streak. This important study reveals that selective breeding can radically change retinal structure in a very short time scale. Given that retinal structure is very much dependent on environment (*16*) and is susceptible within a single species (dogs) to genetic manipulation (*47*) and we believe that retinal design and OP map geometry are linked (*17*), it is probable that OP map structure is also highly dependent on visual environment and developmental pressures, i.e., limited body or brain size, etc.

**Fig. 4.**
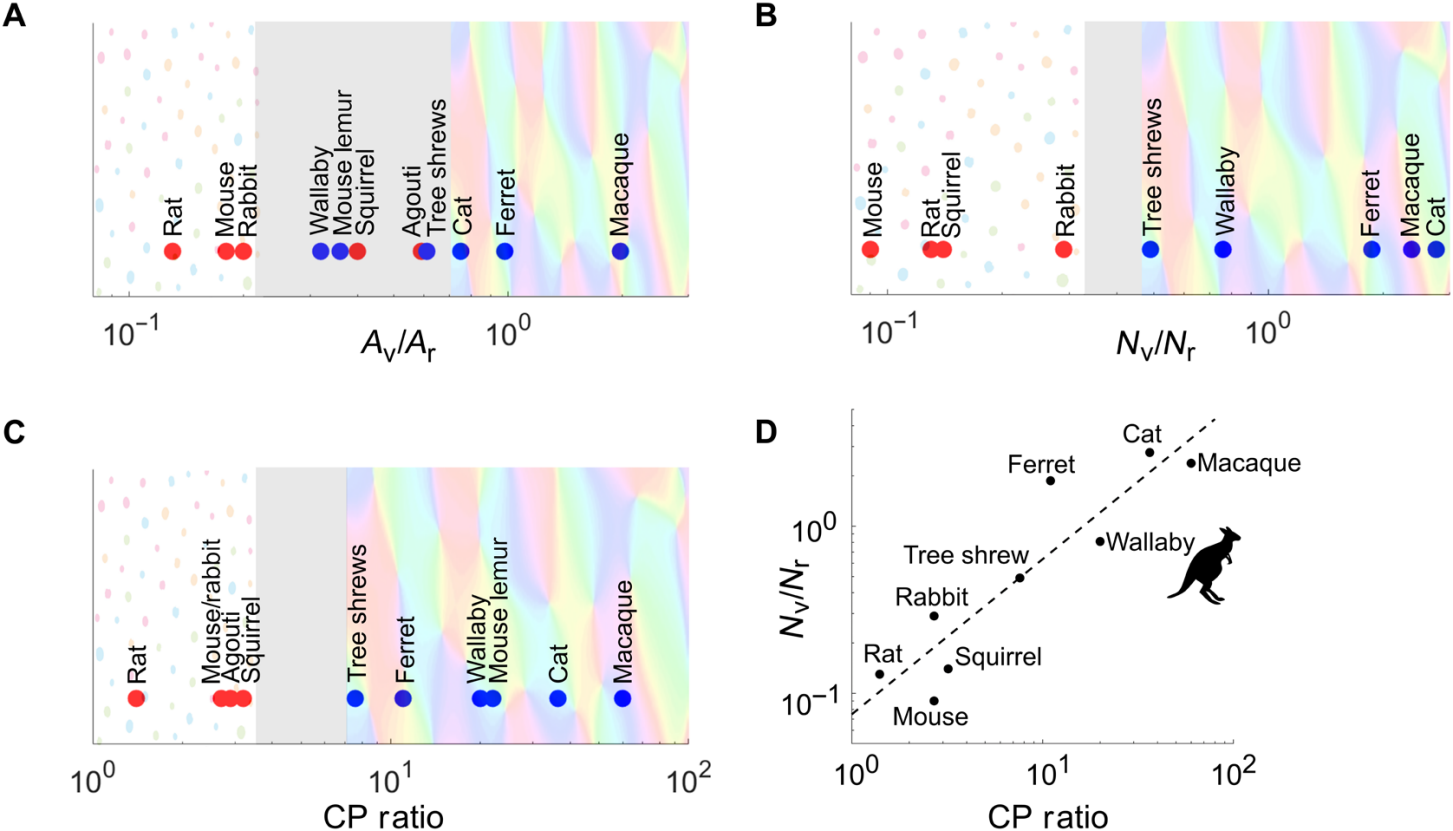
Relationship between retina-to-cortical connections and orientation maps. (**A**) Ratios between the size of V1s and of retinas (*A*_V/_*A*_R_) to predict V1 organization. The red points represent species without pinwheel maps, and the blue points represent species with pinwheel maps. (**B**) Ratios between the size of V1s and of retinas (*N*_V/_*N*_R_) to predict V1 organization. (**C**) Relationship between organization of RGCs and OP map structure. Presence of pinwheels plotted against the CP ratios. For species with a CP ratio of <4 (e.g., rat, mouse, rabbit agouti, and gray squirrel), the pinwheel map was absent. On the other hand, for species with a CP ratio of >7 (e.g., tree shrew, ferret, wallaby, mouse lemur, cat, and macaque), the pinwheel map was present. (**D**) For each species, CP ratios are plotted against the ratios between the number of V1 neurons and RGCs (*N*_V_/*N*_R_) to predict V1 organization. There is a strong linear correlation between CP ratios and (*N*_V_/*N*_R_) (*r*^2^ = 0.71, *P* = 0.001). (Data are in table S1).

We developed a theory (*17*) that cortical OP map structure might be directly related to retinal structure. Using the limited existing data where retinal and cortical structures are known in the same species, we found that the CP ratio, which is the ganglion cell density at the peak or center (C) of the retinal specialization divided by the peripheral (P) retinal density, accurately predicts the type of OP map. Figure 4 illustrates the relationship between CP ratios and the OP map design. In species with CP ratios of <4, pinwheel maps are absent, but for species with CP ratios of >7, pinwheel maps are present. No species with a CP ratio between 4 and 7 has yet been studied. The problem as it stands is that, so far, all mammals with salt-and-pepper OP maps are rodents or rabbits with little retinal specialization, while those with pinwheel OP maps tend to be sophisticated species with complex retinal specializations and binocular lifestyles (e.g., arboreal or predatory). In our view, if a link between retinal and cortical design exists (CP theory; Fig. 4C), then it is also likely that cortical design will have been highly influenced by the environments in which those visual systems have evolved.

The dedication of retinal resources to central vision in some species increases the percentage of RGCs projecting to the lateral geniculate nucleus (LGN), which forms the relay between retina and cortex (*48*). The increased number of LGN connections leads to an overexpansion of V1, which increases exponentially with the number of geniculate afferents (*48*–*49*). In a V1 that has experienced exponential overexpansion, it has been suggested that V1 cells with the same orientation tuning are more likely to cluster together (to improve processing efficiency) (*50*). In agreement with this notion, we have previously noted that high densities of RGCs in the central retina versus the periphery (CP ratio) correlate with the existence of cortical pinwheels (*17*). The wallaby has a CP ratio of 20 and, as with all species studied so far with CP ratios of >4 (*4*–*8*), wallabies have pinwheels in V1. We have also confirmed with the two most recently studied species, agouti (CP ratio = 2.9) and mouse lemur (CP ratio = 22), that the CP ratio can predict the cortical layouts (*18*, *38*). We suggest that there is an all-or-nothing retinal density rule dividing those with or without V1 pinwheel structures (Fig. 4A). In concurrence, recent investigations have shown that when RGCs are sampled by a large number of V1 neurons, the neighboring V1 neurons have highly overlapping RGC RFs, which is correlated with pinwheel structures in V1 (*48*–*49*). To make this assessment, Jang *et al*. (*51*) calculated the ratios of the number (*N*) of V1 neurons to RGCs (*N*_V_/*N*_R_) and of the area (*A*) of V1 to retina (*A*_V_/*A*_R_). Jang *et al*. (*51*) used data from prior studies to calculate the ratios from eight species (see table S1 for primary sources). Following the same procedures, we have added three new species to this analysis (agouti, mouse lemur, and wallaby) to create Fig. 4A. The addition of the new data to this analysis shows that the *A*_V_/*A*_R_ ratio is not a good predicator of cortical layout.

However, the *N*_V_/*N*_R_ ratio may be a good predictor of cortical layout, with the wallaby data fitting within the columnar organization boundary (Fig. 4B). Unfortunately, the V1 and retinal cell densities of agouti and mouse lemur were not available for the plot. We also found a strong linear correlation between CP ratio and both *N*_V_/*N*_R_ (*r*^2^ = 0.71, *P* = 0.001; Fig. 4D). The wallaby and other species with high CP ratios have high retinocortical mapping ratios (RC ratios) and pinwheel OP maps in V1. In contrast, species with low CP ratios and low RC ratios have salt-and-pepper maps. Our data from wallabies support the theory that retinal density gradients, which are generally correlated with the environmental needs of the species in question (*16*), are also closely related to the type of orientation maps in V1.

### Quantifying OP maps

**Fig. 5.**
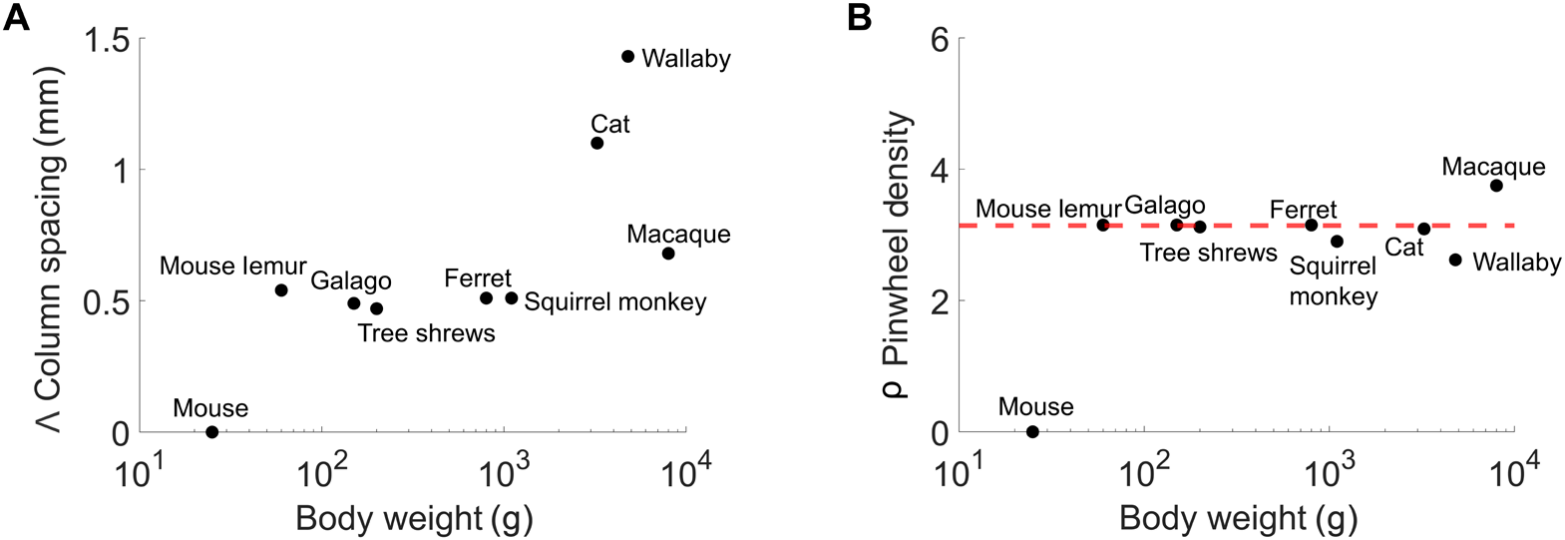
Comparison of column spacing and pinwheel density of species with known OP map architecture. (**A**) The mean spacing (in millimeters) between areas responding to the same orientation from all species with known OP maps, including in the wallaby V1 (1.43 ± 0.40 mm, means ± SD, *n* = 6). (**B**) The mean pinwheel density of all the species with measured OP maps including in the wallaby V1 (2.62 ± 0.40, means ± SD, *n* = 6) (data are in table S2). The red dashed line indicates π.

It has been previously demonstrated that pinwheel density is predicted to be close to the mathematical constant π (~3.14) in models for the joint formation of the system of orientation domains and intracortical circuitry (*9*). The equation used is ρ = ρˆΛ^2^, where Λ^2^ is the area of an orientation hypercolumn. This prediction has been confirmed across several eutherian species (e.g., tree shrews, ferrets, galago, and mouse lemurs). Data are in the table S2. We used the same algorithm on the spatial organization of orientation domains and pinwheels in the central and peripheral field representation of the tammar wallaby. We found a mean pinwheel density of 2.62 ± 0.40 for wallaby cortex (means ± SD, *n* = 6), which is less than π and lower than the cat cortex (ρ =3.09; Fig. 5B) (*52*). The mean hypercolumn spacing for wallaby cortex (1.43 ± 0.40 mm, means ± SD, *n* = 6) was also similar to that reported in the cat (1.10 mm) (Fig. 5A).

While radially arranged maps are the most commonly described form, we found differences in the organization of OP in the central and peripheral visual field representations of wallaby visual cortex. We observed iso-orientation domains arranged radially around pinwheels in the OP maps for the central visual field. In contrast, in the peripheral representation, we found the coexistence of radial and linear organization of iso-orientation domains. Vertical and horizontal OPs were organized linearly and alternately, parallel to the medial-lateral axis of the cortical surface. Pinwheel centers were found at the edges of these linear bands. A very similar type of mixed radial linear orientation map has been reported on the boundaries between areas 17 and 18 in cat, ferret, and tree shrew (*5*, *6*, *53*) and in an area 18 of cat, showing that variations to the basic radial pinwheel map exist in multiple brain areas and species.

Given that mixed radial linear maps are often found on the border between areas 17 and 18, it has been suggested that “this border might induce a boundary condition that biases the organization for orientation in favor of linearity” (*53*). In the wallaby, the mixed radial linear arrangement is found in the medial regions of area 17 quite some distance from the area 17/18 border. Hence, the peripheral field representation in area 17 of wallaby resembles area 18 in cat and is not related to the proximity to the 17/18 border. It remains to be seen whether the radial linear organization of the map in wallaby cortex is functionally relevant, perhaps related to viewing of the peripheral visual field during forward locomotion.

We argue that pinwheel OP maps might be the ancestral design, as they are present in all mammalian branches studied so far except the recently evolved Glires. It might be a case of evolution, switching off the pinwheel gene for the rodents and rabbits, as they may have become redundant in their environment. It might also be that the Glires have reached their innate developmental boundaries (i.e., retinal and cortical cell counts) and are physically restricted to forming salt-and-pepper maps. Our findings from wallaby cortex suggest that the required genetics for pinwheel formation are likely present in all mammals but are only revealed when the visual ecology of a species demands concentration of retinal sampling (high CP ratio) and associated divergence of connectivity from the retina to cortex (high RC ratio).

## MATERIALS AND METHODS

### Wallabies

Experiments were conducted on six adult tammar wallabies (weighing 3.9 to 5.7 kg) at the National Vision Research Institute per the National Health and Medical Research Council’s Australian Code of Practice for the Care and Use of Animals for Scientific Purposes. The Animal Ethics Committee at the University of Melbourne approved all the experimental procedures (approval ID: 1714178).

### Surgical procedure

Following procedures previously described by Jung (*54*), ketamine (10 mg/kg), medetomidine (0.015 mg/kg), and methadone (0.4 mg/kg) were intramuscularly administered to the wallaby to induce anesthesia. A 22-gauge over-the-needle catheter was placed in one of the cephalic veins, and an orotracheal tube was placed and connected to a pediatric rebreathing system. Isoflurane (1 to 2%) in a mixture of 50% oxygen and 50% nitrogen (1 to 2 liter/min) was administered to maintain anesthesia throughout the surgery. The wallaby was mechanically ventilated to maintain an end tidal carbon dioxide between 30 and 45 mmHg. Fluid therapy consisted of continuous intravenous infusion of Hartmann’s solution (50% by volume) and 0.9% NaCl solution (50% by volume) at a rate of 2.5 ml/kg per hour. The animal’s rectal temperature was maintained between 36° and 37.5°C via a feedback-controlled heating blanket. The head of the animal was stabilized with a stereotaxic frame and custom-made ear bars.

At the start of each experiment, the animal was given lincomycin (10 mg/kg) and spectinomycin (20 mg/kg) intramuscularly and paraffin oil (10 ml) orally to reduce intestinal bloating during prolonged anesthesia. During surgery, the animals were intramuscularly given phytomenadione (10 mg/kg) and tranexamic acid (100 mg/kg) to counter any bleeding. The animal also received daily intramuscular injections of atropine (0.2 mg/kg), dexamethasone phosphate (1.5 mg/kg), and Clavulox (0.05 ml/kg) to reduce salivation, prevent cerebral oedema, and control infection, respectively.

Using published stereotaxic coordinates (*55*) and anatomical markers for area 17 (V1), we created a small craniotomy (approximately 10 mm by 10 mm) over the left hemisphere of V1. The craniotomy extended from 15 to 25 mm posterior to bregma and 4 to 14 mm lateral from the midline. To expose more of the central visual field, in two experiments, we adjusted the craniotomy to extend from 7 to 17 mm posterior to bregma and 6 to 16 mm lateral from the midline (fig. S1, B to G). Normative topographical mapping of wallaby V1 (*25*) was used as a guide (fig. S1A). A durotomy was performed to expose the surface of the cortex, and a stainless steel chamber was affixed to the skull with dental cement.

After surgery, anesthesia was maintained with halothane (0.5%) in a 2:1 mixture of nitrous oxide and oxygen. Atropine sulfate eye drops (1%) were administered daily to maintain pupil dilation and to retract the nictitating membranes. To eliminate eye movements, neuromuscular blockade was achieved using a bolus of vecuronium bromide (0.05 mg/kg, intravenously), followed by continuous intravenous infusion of vecuronium (0.1 mg/kg per hour). The vecuronium was diluted in a Hartmann’s solution containing 5% glucose. Zero-power gas-permeable contact lenses were fitted to protect the corneas. Refractive errors were measured using retinoscopy and corrected with spherical lenses placed in front of the eyes to focus the stimuli on the retina. At the conclusion of the experiment, animals were euthanized with an intravenous injection of an overdose of barbiturate (sodium pentobarbital; 150 mg/kg).

### Visual stimuli

Visual stimuli were generated with a ViSaGe visual stimulus generator (Cambridge Research Systems, Cambridge, UK) and displayed on a calibrated, gamma-corrected liquid crystal display monitor (ASUS VG248QE, 1920 pixels by 1080 pixels, refresh rate of 60 Hz, and 1-ms response time) at a viewing distance of 30 cm. The monitor was placed in two positions, one central to stimulate the central visual field and the other peripheral (45° from the central position) to stimulate the peripheral visual field. Visual stimuli for optical imaging were luminance-defined oriented square-wave gratings (0.15 cycles/deg; Michelson contrast: 100%) presented within a rectangular aperture (60° by 53°, 53.1 cm by 29.9 cm). Gratings drifted (temporal frequency of 2 Hz) in one of the 16 directions (orthogonal to their orientation) equally spaced between 0° and 360°. Each stimulus direction was presented 30 times, in a block-randomized random order [each block consisted of eight grating stimuli, drifting in eight different directions, together with a blank (no grating) condition].

For electrophysiology, white Gaussian noise (WGN) stimuli were used to estimate the spatial RFs of cortical cells. WGN images composed of 90 pixels by 90 pixels over 53° of the visual field, with its mean pixel value matched to the mean luminance of the display monitor. If the mean spatial RF size for a track was greater than 3° of visual field (refer to fig. S4), then the size of WGN images was adjusted to 60 pixels by 60 pixels.

Noise stimuli were presented in blocks, each composed of 12,000 WGN images. Each image was presented for 0.033 seconds, followed by a blank screen at the mean luminance, and presented for the same duration. The SD of each noise block was determined to obtain 90% of pixels with nonsaturated values between 0 and 1, while the remaining pixels were clipped to the maximum or minimum luminance (1.0 and 0.0, respectively). Several noise blocks were presented per experiment.

For electrophysiology, in addition to the WGN stimuli, we used drifting sinusoidal gratings with a circular aperture (60° diameter) set to high contrast (Michelson contrast: 100%) on a gray background at the mean screen luminance. We presented various combinations of spatial and temporal frequencies to determine a cell’s preferred direction (i.e., 16 directions equally spaced between 0° and 337.5°). Grating stimuli were presented in random order, drifting for at least 1 s, for at least five trials.

### Optical imaging and data acquisition

For imaging, the cranial chamber was filled with silicone oil (Dow Corning 200, 50 cSt) and sealed with a glass coverslip to stabilize the cortex. We imaged the exposed area using a high-sensitivity charge-coupled device camera (Teledyne DALSA, Waterloo, ON, Canada) fitted with a tandem lens macroscope consisting of two Nikkor 50 mm f/1.2 lenses. The camera was configured to bin pixels of 2 by 2, resulting in an image resolution of 512 pixels by 512 pixels (1 pixel = 24 μm square). For imaging, the focal plane of the camera was positioned 800 to 1000 μm below the surface vasculature using a micromanipulator.

A custom-built light-emitting diode light source (Agilent Technologies, HSMQ-C150) was used to epi-illuminate the surface of the cortex with green light (540 nm). Previous studies have used red light (wavelength > 600 nm) for intrinsic signal imaging, but as suggested in an earlier report (*56*), we used green light (540 nm) to obtain a larger signal-to-noise ratio. The component of the intrinsic signal that we could detect using green light was likely the localized blood volume in active regions of the cortex. Regions with large blood volumes or high deoxyhemoglobin concentration as a consequence of increased levels of neural activity exhibit less cortical tissue reflectance, and the resulting image appears darker (*24*). The green light revealed blood vessel artifacts on some occasions, which were mitigated by extended spatial decorrelation (ESD; see “Map generation” section below and fig. S2).

We imaged cortical responses during presentation of visual stimuli (as described above). Images were acquired continuously at a rate of 5 Hz for 10 s for each stimulus condition. The image acquisition started 2 s before the stimulus onset. Image acquisition was synchronized to the phase of the respirator (i.e., each trial commenced at maximum inspiration). Gratings were presented for 5 s followed by a recovery period of at least 3 s during which the monitor displayed an isoluminant gray screen with luminance equal to the mean luminance of the gratings.

### Map generation

MATLAB was used to process and analyze all optical imaging data. The raw images were spatially cropped to the cortical region of interest. For each trial, images acquired before the stimulus onset (i.e., reference images) were summed and subtracted from all subsequent images to remove any stimulus-independent patterns across images (*8*). Trials from opposite directions of stimulus motion were averaged to obtain eight orientation conditions, and trials from 16 directions of stimulus motion were used to obtain 16 direction conditions. For each stimulus condition, we averaged difference images across all trials. To remove baseline activity and any uneven illumination, we subtracted from each of the trial averages a “cocktail blank” obtained by summing the responses from all stimulus conditions (*57*).

Each of the imaging frames were high-pass Gaussian–filtered (σ = 20 pixels = 480 μm) to remove the large-scale changes in illumination across the images (*56*). The images were then low-pass Gaussian–filtered (σ = 2 pixels = 48 μm) to fulfill the requirement of the ESD method, which assumes the sources to be smoothed. The signs of the reflectance values were reversed because a decrease in reflectance corresponds to an increase in neural activity.

#### *Extended spatial decorrelation*

The ESD method described by Schießl *et al*. (*57*) was used to generate the feature maps. In principle, the ESD method relies on the second-order statistics of the data to convert individual imaging frames into separable components from different sources and ultimately separate stimulus-specific intrinsic signals from biological noise and artifacts. Assuming that source components are spatiotemporally separable, the changes in reflectance patterns can be written as

$$S_{j}(r,t)=a_{j}(t)s_{j}(r),j=1,\ldots ,N$$

where *r* represents the reflectance change; *t* represents time; *s_j_*(*r*) is the spatial pattern of the source, *s_j_*, which is constant at all times for each frame; and *j* and *a_j_*(*t*) represents the amplitude of the source, *s_j_*. The overall optical imaging dataset can be described as

$$x_{m}(r)=\sum_{j=1}^{N}a_{\mathrm{mj}}s_{j}(r)+n_{m}(r)$$

where *a_mj_* is the amplitude in the *m*th imaging frame, which gives rise to the time course of the source (*s_j_*), and *n_m_*(*r*) is the sensor noise produced during data collection (i.e., photon shot noise and camera readout noise). If the coefficients *a_mj_* are combined in the form of a mixing matrix [A = (*a_mj_*)], then the statistical data model can be described as

x(r)=As(r)+n(r)

where ***x***(*r*) is the pixel time series, which represents a single data point in the mixture space, *s*(*r*) is the spatial component of the sources in the source vector, *s*_1_(*r*), …, *s_N_*(*r*), and *n*(*r*) is the sensor noise.

The aim of the ESD method is to find the demixing matrix W, which can effectively reconstruct the sources from their noisy mixtures, $\hat{s}(r)=Wx(r)$, from a statistical independence criterion on the original sources. This assumption is that the sources are temporally and spatially uncorrelated with each other. The cross-correlation function used to decorrelate the two source patterns *s_l_*(*r*) and *s_m_*(*r*) can be written as

$$C_{s}^{\mathrm{lm}}(∆r)=\frac{1}{Q}\sum_{r}s_{l}(r)s_{m}(r+∆r)$$

where the shift ∆*r* is often called the lag of the correlation function and each lag ∆*r* delivers one value $C_{s}^{\mathrm{lm}}(\text{∆}r)$, and, as the number of overlapping pixels between *s_l_* and *s_m_* decreases with the size of ∆*r*, we normalize with the value *Q*, which is the number of pixels the two sources still have in common.

As a first step in ESD, different sources *s_l_* and *s_m_* are decorrelated in time from each other using the zero-shift correlation matrix of ***C***_s_(0)

$$y(r)=\Lambda_{0}^{-1/2}\;V_{0}^{T}\;x(r)$$

where ***y***(*r*) is the decorrelated signal, **Λ**_0_ is a diagonal matrix with the eigenvalues of ***C***_s_(0) along the diagonal, and $V_{0}^{T}$ is the matrix of corresponding eigenvectors arranged in rows. This transforms the sources to have identity covariance. Second, the sources *s_l_* are decorrelated in space by using shifted versions of *s_m_*; i.e., $C_{s}^{\mathrm{lm}}(\text{∆}r)$ vanished for all ∆*r*. A shift of ∆*r* = (5,5) pixels was used to perform the single shift ESD. This decorrelation matrix, *U*, is the solution of the eigenvalue equation

$$(C_{s}(∆r)+C_{s}(-∆r))U=\Lambda_{∆r}\;U$$

The overall demixing matrix, W, was obtained by multiplying the decorrelation derived from the zero-shift correlation matrix, $C_{s}^{\mathrm{ml}}(0)$, and the second decorrelation matrix derived from $C_{s}^{\mathrm{lm}}(\text{∆}r)$

$$s(r)=U^{-1}\Lambda_{0}^{-1/2}\;V_{0}^{T}\;x(r)$$

**Fig. 6.**
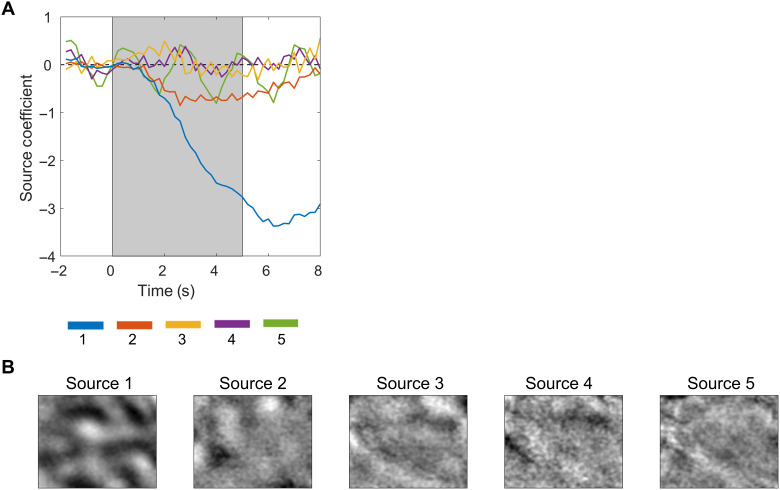
ESD separates mapping signal from green light imaging. (**A**) Time course of the separated components (sources 1 to 5). The gray shaded region shows the stimulus period (5 s). It is clear that source 1 is the mapping signal as the time course represents the change in absorption during the presentation of visual stimuli. (**B**) The corresponding reconstructed sources.

The demixing matrix, W, was then applied to the noisy mixture to generate 50 decorrelated signal components. The time course of the coefficients (i.e., which is the inverse of the demixing matrix) derived from each source was plotted to determine which of the sources corresponded to stimulus presentation (Fig. 6). In most cases, the correct source was obvious, as the time series resembled the typical signal from the intrinsic optical imaging response (i.e., a rise at the time of stimulus onset and maximal at the stimulus offset). Figure 6B illustrates the five decorrelated sources corresponding to plots in Fig. 6A. It is clear from the dark patches present in the first source that it is the correct one corresponding to the stimulus-specific intrinsic signal, and the rest of the sources are most likely due to biological noise or blood vessel artifacts. Each frame of the correct source was multiplied by its corresponding coefficient as an estimate of the response signal in the data, and the map for each stimulus condition was created by averaging the final five stimulus frames (i.e., frames with the highest signal). We removed any remaining high-frequency noise caused by the remaining blood vessel artifacts by applying a low-pass Gaussian filter (σ = 8 pixels = 192 μm).

### OP maps

To visualize the organization of OPs across the visual cortex, monocular OP maps were generated by vectorially summing the supplied single condition maps (*8*). The two vector components of the OP map were obtained by summing the responses to eight stimulus conditions (i.e., orientations) for each pixel. Let *R*_θ, *R*_ be the sum of responses to orientation θ produced by the right eye

$$\;\text{real}(\text{OP})=\sum_{\theta }(R_{\theta ,R})\text{cos}(2\theta )$$

$$\text{imag}(\text{OP})=\sum_{\theta }(R_{\theta ,R})\text{sin}(2\theta )$$

$$\theta_{\text{Pref}}=a\text{tan}2(x+\mathrm{jy})/2$$

where θ values in the sum were 0°, 22.5°, …, and 157.5°. For each pixel, the angle of the resulting complex number was converted back to orientation between 0° and 180° by dividing by 2 and color-coded.

### Direction preference maps

The layout of direction preferences across the cortex was determined by vectorially summing the single condition maps obtained from 16 directions. The two vector components of the direction preference (DP) map were obtained by summing the responses to 16 stimulus conditions for each pixel. Let *R*_θ, *R*_ be the sum of responses to direction θ produced by the right eye

$$\text{real}(\text{DP})=\sum_{\theta }(R_{\theta ,R})\text{cos}(\theta )$$

$$\text{imag}(\text{DP})=\sum_{\theta }(R_{\theta ,R})\text{sin}(\theta )$$

$$\theta_{\text{Pref}}=a\text{tan}2(x+\mathrm{jy})$$

where θ values in the sum were 0°, 22.5°, …, and 337.5°. For 2 pixels by 2 pixels, the angle of the resulting vector was indicated by the direction of arrows.

### Analysis of OP maps

For all the OP maps obtained, each pixel was binned according to its preferred orientation into 22.5° wide OP bins centered at 11.25°, 33.75°, ..., 168.75°, etc. These orientation bins were set to produce a histogram, which illustrates the proportion of cortical area representing different orientations.

Pinwheel locations were determined using the method previously described by Carreira-Perpinan *et al*. (*58*). For each pixel, the winding number was calculated by summing the increments of orientation angle in [−π/2, π/2] along a closed path of radius equal to 1 pixel and dividing it by 2π. The winding number is 0 for nonpinwheel points and +1/2 or −1/2 for pinwheel points that have orientation angles increasing around the pinwheel in a clockwise or counterclockwise direction, respectively. The exact location of a pinwheel was determined by clustering pixels with nonzero winding numbers and computing their centers of mass. Pinwheel density was calculated by the number of pinwheels per pixel multiplied by the square of the map wavelength (size of hypercolumn). The wavelength was defined as the mean Fourier wavelength of the map averaged over all directions (*9*).

### Electrophysiology recordings

Extracellular recordings were conducted using NeuroNexus 32-channel multielectrode arrays (MEAs). We used two types of MEAs: a single-shank probe (6 mm; 1 × 32 sites spaced at 100-μm intervals) or a four-shank probe (6 mm in length; 4 × 8 sites spaced at 100-μm intervals). For each recording session, only one type of MEA was used. The arrays were vertically inserted into the cortex using a piezoelectric drive (Burleigh inchworm and 6000 controller, Burleigh Instruments, Rochester, NY). Extracellular signals from 32 channels were simultaneously acquired at 30 kHz using a CerePlex acquisition system and Central software (Blackrock Microsystems, Salt Lake City, Utah).

### Spike sorting

Extracellular recordings from MEAs can have neighboring neurons whose spikes are picked up by the same electrode site, and, in other cases, the spikes from some neurons may appear on multiple electrode sites. To separate spikes from different neurons, we used an automatic spike-sorting program called Kilosort (*59*), which is commonly used for sorting recordings from dense arrays.

First, the raw data were high-pass filtered at 300 Hz to remove low-frequency fluctuations such as the local field potential. Then, the median signal from all recording sites was subtracted at each time point to reduce the effect of artifacts found across all channels. This process is described by Ludwig *et al*. (*60*) as common average referencing. Second, the filtered data were whitened across channels to remove spatially correlated noise, which arises from neurons located far from the probe. The whitening decorrelates the signals from each other by having each of the signals’ variance equal to 1 (i.e., identity matrix). Whitening was performed by estimating the whitened matrix with the data that had any of the putative spikes above a threshold criterion removed. The whitened matrix was then multiplied to the data matrix containing all channels. Using a three-dimensional singular value decomposition (SVD) of the spike’s spatiotemporal waveform, each spike in the filtered signal was assigned a principal component. This decomposition method denoised the waveforms and removed any irrelevant channels. The running average waveform derived from SVD was used to obtain the predefined templates. In an iterative process, the algorithm was used to find spike times from the raw data that were similar to the predefined template waveform (template matching). After subtracting the matched spikes, we repeatedly performed template matching to find spatiotemporally overlapping spikes.

We used the geometry of the recording probe to create an adjacency map, which allowed us to detect neighboring sites. We were then able to cluster the spikes, which appeared simultaneously across multiple neighboring sites as one unit. The spike clusters were manually curated for further verification using the graphical user interface phy (*61*). Single units were identified from the presence of well-separated clusters in their feature space and from the profound refractory period in their interspike interval histograms, which confirms that spikes are nonoverlapping.

### Model estimation

We used the nonlinear input model described by Almasi *et al*. (*33*) to estimate the spatial RFs. This framework is an adaptation of the original model, introduced by McFarland *et al*. (*50*), that estimates all model parameters simultaneously.

#### *Orientation selectivity from RF filters*

We quantified the orientation selectivity of every filter with an OB index as follows

$$\text{OB}=\frac{|\sum_{k}R_{k}\;\text{exp}\;(i2\;\theta_{k})|}{\sum_{k}R_{k}\;}$$

where *R_k_* represents the neuronal response at orientation θ*_k_*. We adapted this measure for the amplitude spectrum of filters, and *R_k_* here represents the amplitude spectrum of the filter sampled at orientation θ*_k_*. An OB index of 0 is indicative of a cell responding equally to all orientations, while an OB index of 1 suggests that the cell is responding to only one particular orientation. Hence, orientation selectivity is greater as OB indices increase. We adopted an OB index of 0.2 as the division between oriented and nonoriented RFs (*62*). For single units with more than one filter, the OB index was calculated for all filters, and the minimum OB was used.

To examine the relationship between OPs obtained in OP maps versus single units, we used photographs of surface blood vessels and superimposed OP maps to ascertain within which columns we had placed our electrode arrays. Arrays were placed perpendicular to the cortical surface, and we correlated the orientation tuning of cells in the upper 10 channels (i.e., upper 0.2 to 1 mm). The mean OP from optical imaging of 10 pixels by 10 pixels surrounding the electrode was calculated and compared with the OPs of the units recorded at that location, as described by Cloherty *et al*. (*56*). The measures OP from single unit recordings correlated strongly with the OP maps (*r*^2^ = 0.676, *P* = 0.001; Fig. 3B), with a median absolute difference of 8°.

## Supplementary Materials

### This PDF file includes:

Figs. S1 to S6 Tables S1 and S2 References

## Notes

View/request a protocol for this paper from *Bio-protocol*.

## References

[1] J. Kremkow, J. Jin, Y. Wang, J. M. Alonso, Principles underlying sensory map topography in primary visual cortex. Nature 533, 52–57 (2016). 27120164 10.1038/nature17936 PMC4860131

[2] J. Cang, R. C. Rentería, M. Kaneko, X. Liu, D. R. Copenhagen, M. P. Stryker, Development of precise maps in visual cortex requires patterned spontaneous activity in the retina. Neuron 48, 797–809 (2005). 16337917 10.1016/j.neuron.2005.09.015 PMC2562716

[3] F. Briggs, Organizing principles of cortical layer 6. Front. Neural Circuits 4, 3 (2010). 20179784 10.3389/neuro.04.003.2010 PMC2826182

[4] T. Bonhoeffer, A. Grinvald, Iso-orientation domains in cat visual cortex are arranged in pinwheel-like patterns. Nature 353, 429–431 (1991). 1896085 10.1038/353429a0

[5] B. Chapman, M. P. Stryker, T. Bonhoeffer, Development of orientation preference maps in ferret primary visual cortex. J. Neurosci. 16, 6443–6453 (1996). 8815923 10.1523/JNEUROSCI.16-20-06443.1996 PMC2669086

[6] W. H. Bosking, Y. Zhang, B. Schofield, D. Fitzpatrick, Orientation selectivity and the arrangement of horizontal connections in tree shrew striate cortex. J. Neurosci. 17, 2112–2127 (1997). 9045738 10.1523/JNEUROSCI.17-06-02112.1997 PMC6793759

[7] D. Hubel, T. Wiesel, Anatomical demonstration of columns in the monkey striate cortex. Nature 221, 747–750 (1969). 4974881 10.1038/221747a0

[8] G. G. Blasdel, Orientation selectivity, preference, and continuity in monkey striate cortex. J. Neurosci. 12, 3139–3161 (1992). 1322982 10.1523/JNEUROSCI.12-08-03139.1992 PMC6575662

[9] M. Kaschube, M. Schnabel, S. Löwel, D. M. Coppola, L. E. White, F. Wolf, Universality in the evolution of orientation columns in the visual cortex. Science 330, 1113–1116 (2010). 21051599 10.1126/science.1194869 PMC3138194

[10] D. B. Chklovskii, A. A. Koulakov, Maps in the brain: What can we learn from them? Annu. Rev. Neurosci. 27, 369–392 (2004). 15217337 10.1146/annurev.neuro.27.070203.144226

[11] K. Ohki, S. Chung, Y. H. Ch’ng, P. Kara, R. C. Reid, Functional imaging with cellular resolution reveals precise micro-architecture in visual cortex. Nature 433, 597–603 (2005). 15660108 10.1038/nature03274

[12] S. D. Van Hooser, J. A. F. Heimel, S. Chung, S. B. Nelson, L. J. Toth, Orientation selectivity without orientation maps in visual cortex of a highly visual mammal. J. Neurosci. 25, 19–28 (2005). 15634763 10.1523/JNEUROSCI.4042-04.2005 PMC6725193

[13] D. N. Ferreiro, S. A. Conde-Ocazionez, J. H. N. Patriota, L. C. Souza, M. F. Oliveira, F. Wolf, K. E. Schmidt, Spatial clustering of orientation preference in primary visual cortex of the large rodent agouti. Iscience 24, 101882 (2021). 33354663 10.1016/j.isci.2020.101882 PMC7744940

[14] D. L. Ringach, P. J. Mineault, E. Tring, N. D. Olivas, P. Garcia-Junco-Clemente, J. T. Trachtenberg, Spatial clustering of tuning in mouse primary visual cortex. Nat. Commun. 7, 1–9 (2016). 10.1038/ncomms12270 PMC4974656 27481398

[15] S. Kondo, T. Yoshida, K. Ohki, Mixed functional microarchitectures for orientation selectivity in the mouse primary visual cortex. Nat. Commun. 7, 13210 (2016). 27767032 10.1038/ncomms13210 PMC5078743

[16] A. Hughes, in *The Visual System in Vertebrates* (Springer, 1977), pp. 613–756.

[17] M. Ibbotson, Y. J. Jung, Origins of functional organization in the visual cortex. Front. Syst. Neurosci. 14, 10 (2020). 32194379 10.3389/fnsys.2020.00010 PMC7063058

[18] C. L. A. Ho, R. Zimmermann, J. D. F. Weidinger, M. Prsa, M. Schottdorf, S. Merlin, T. Okamoto, K. Ikezoe, F. Pifferi, F. Aujard, A. Angelucci, F. Wolf, D. Huber, Orientation preference maps in *Microcebus murinus* reveal size-invariant design principles in primate visual cortex. Curr. Biol. 31, 733–741.e7 (2021). 33275889 10.1016/j.cub.2020.11.027 PMC9026768

[19] K. E. Schmidt, F. Wolf, Punctuated evolution of visual cortical circuits? Evidence from the large rodent *Dasyprocta leporina*, and the tiny primate *Microcebus murinus*. Curr. Opin. Neurobiol. 71, 110–118 (2021). 34823047 10.1016/j.conb.2021.10.007

[20] W. Keil, M. Kaschube, M. Schnabel, Z. F. Kisvarday, S. Löwel, D. M. Coppola, L. E. White, F. Wolf, Response to comment on “universality in the evolution of orientation columns in the visual cortex”. Science 336, 413–413 (2012). 10.1126/science.1194869 PMC3138194 21051599

[21] M. Kaschube, Neural maps versus salt-and-pepper organization in visual cortex. Curr. Opin. Neurobiol. 24, 95–102 (2014). 24492085 10.1016/j.conb.2013.08.017

[22] Z.-X. Luo, C.-X. Yuan, Q.-J. Meng, Q. Ji, A Jurassic eutherian mammal and divergence of marsupials and placentals. Nature 476, 442–445 (2011). 21866158 10.1038/nature10291

[23] K. W. Ashwell, Anterior commissure versus corpus callosum: A quantitative comparison across mammals. Fortschr. Zool. 119, 126–136 (2016). 10.1016/j.zool.2016.02.004 26961186

[24] A. Grinvald, E. Lieke, R. D. Frostig, C. D. Gilbert, T. N. Wiesel, Functional architecture of cortex revealed by optical imaging of intrinsic signals. Nature 324, 361–364 (1986). 3785405 10.1038/324361a0

[25] T. Vidyasagar, J. Wye-Dvorak, G. Henry, R. Mark, Cytoarchitecture and visual field representation in area 17 of the tammar wallaby (*Macropus eugenii*). J. Comp. Neurol. 325, 291–300 (1992). 1281175 10.1002/cne.903250211

[26] M. Ibbotson, R. Mark, Orientation and spatiotemporal tuning of cells in the primary visual cortex of an Australian marsupial, the wallaby *Macropus eugenii*. J. Comp. Physiol. A 189, 115–123 (2003). 10.1007/s00359-002-0379-6 12607040

[27] M. Weliky, W. H. Bosking, D. Fitzpatrick, A systematic map of direction preference in primary visual cortex. Nature 379, 725–728 (1996). 8602218 10.1038/379725a0

[28] C. Rocha-Miranda, R. Linden, E. Volchan, R. Lent, R. Bombardieri Jr., Receptive field properties of single units in the opossum striate cortex. Brain Res. 104, 197–219 (1976). 816419 10.1016/0006-8993(76)90614-4

[29] J. C. Dooley, M. S. Donaldson, L. A. Krubitzer, Cortical plasticity following stripe rearing in the marsupial *Monodelphis domestica*: Neural response properties of V1. J. Neurophysiol. 117, 566–581 (2017). 27852732 10.1152/jn.00431.2016 PMC5288476

[30] D. P. Crewther, S. G. Crewther, K. J. Sanderson, Primary visual cortex in the brushtailed possum: Receptive field properties and corticocortical connections. Brain Behav. Evol. 24, 184–197 (1984). 6093921 10.1159/000121316

[31] D. H. Hubel, T. N. Wiesel, Receptive fields, binocular interaction and functional architecture in the cat’s visual cortex. J. Physiol. 160, 106–154 (1962). 14449617 10.1113/jphysiol.1962.sp006837 PMC1359523

[32] D. H. Hubel, T. N. Wiesel, Receptive fields and functional architecture of monkey striate cortex. J. Physiol. 195, 215–243 (1968). 4966457 10.1113/jphysiol.1968.sp008455 PMC1557912

[33] A. Almasi, H. Meffin, S. L. Cloherty, Y. Wong, M. Yunzab, M. R. Ibbotson, Mechanisms of feature selectivity and invariance in primary visual cortex. Cereb. Cortex 30, 5067–5087 (2020). 32368778 10.1093/cercor/bhaa102

[34] J. Stone, B. Dreher, A. Leventhal, Hierarchical and parallel mechanisms in the organization of visual cortex. Brain Res. Rev. 1, 345–394 (1979). 10.1016/0165-0173(79)90010-9 231475

[35] S. D. Van Hooser, Similarity and diversity in visual cortex: Is there a unifying theory of cortical computation? Neuroscientist 13, 639–656 (2007). 17911223 10.1177/1073858407306597

[36] S. Chenchal Rao, L. J. Toth, M. Sur, Optically imaged maps of orientation preference in primary visual cortex of cats and ferrets. J. Comp. Neurol. 387, 358–370 (1997). 9335420 10.1002/(sici)1096-9861(19971027)387:3<358::aid-cne3>3.3.co;2-v

[37] P. Clarke, I. Donaldson, D. Whitteridge, Binocular visual mechanisms in cortical areas I and II of the sheep. J. Physiol. 256, 509–526 (1976). 1271290 10.1113/jphysiol.1976.sp011336 PMC1309322

[38] Y. E. Zhang, P. Landback, M. D. Vibranovski, M. Long, Accelerated recruitment of new brain development genes into the human genome. PLOS Biol. 9, e1001179 (2011). 22028629 10.1371/journal.pbio.1001179 PMC3196496

[39] D. L. Silver, Genomic divergence and brain evolution: How regulatory DNA influences development of the cerebral cortex. Bioessays 38, 162–171 (2016). 26642006 10.1002/bies.201500108 PMC4718859

[40] S. Collin, in *Adaptive Mechanisms in the Ecology of Vision* (Springer, 1999), pp. 509–535.

[41] S. P. Collin, A web-based archive for topographic maps of retinal cell distribution in vertebrates. Clin. Exp. Optom. 91, 85–95 (2008). 18045254 10.1111/j.1444-0938.2007.00228.x

[42] C. W. Oyster, E. S. Takahashi, D. C. Hurst, Density, soma size, and regional distribution of rabbit retinal ganglion cells. J. Neurosci. 1, 1331–1346 (1981). 7320749 10.1523/JNEUROSCI.01-12-01331.1981 PMC6564131

[43] A. Shinozaki, Y. Hosaka, T. Imagawa, M. Uehara, Topography of ganglion cells and photoreceptors in the sheep retina. J Comp Neurol 518, 2305–2315 (2010). 20437529 10.1002/cne.22333

[44] A. Navarro-Sempere, Y. Segovia, M. García, Comparative analysis of retinal ganglion cell topography and behavioral ecology in Australian marsupials. Int. J. Morphol. 36, 248–257 (2018).

[45] F. Knolle, R. P. Goncalves, A. J. Morton, Sheep recognize familiar and unfamiliar human faces from two-dimensional images. R. Soc. Open Sci. 4, 171228 (2017). 29291109 10.1098/rsos.171228 PMC5717684

[46] U. Drager, J. Olsen, Ganglion-cell distribution in the retina of the mouse. Invest. Ophthalmol. Vis. Sci. 20, 285–293 (1981). 6162818

[47] P. McGreevy, T. D. Grassi, A. M. Harman, A strong correlation exists between the distribution of retinal ganglion cells and nose length in the dog. Brain Behav. Evol. 63, 13–22 (2004). 14673195 10.1159/000073756

[48] J. Kremkow, J.-M. Alonso, Thalamocortical circuits and functional architecture. Annu. Rev. Vision Sci. 4, 263–285 (2018). 10.1146/annurev-vision-091517-034122 PMC7525828 29856937

[49] C. F. Stevens, An evolutionary scaling law for the primate visual system and its basis in cortical function. Nature 411, 193–195 (2001). 11346795 10.1038/35075572

[50] J. M. McFarland, Y. Cui, D. A. Butts, Inferring nonlinear neuronal computation based on physiologically plausible inputs. PLOS Comput. Biol. 9, e1003143 (2013). 23874185 10.1371/journal.pcbi.1003143 PMC3715434

[51] J. Jang, M. Song, S.-B. Paik, Retino-cortical mapping ratio predicts columnar and salt-and-pepper organization in mammalian visual cortex. Cell Rep. 30, 3270–3279.e3 (2020). 32160536 10.1016/j.celrep.2020.02.038

[52] M. Schottdorf, W. Keil, D. Coppola, L. E. White, F. Wolf, Random wiring, ganglion cell mosaics, and the functional architecture of the visual cortex. PLOS Comput. Biol. 11, e1004602 (2015). 26575467 10.1371/journal.pcbi.1004602 PMC4648540

[53] A. Shmuel, A. Grinvald, Functional organization for direction of motion and its relationship to orientation maps in cat area 18. J. Neurosci. 16, 6945–6964 (1996). 8824332 10.1523/JNEUROSCI.16-21-06945.1996 PMC6579248

[54] Y. J. Jung, thesis, University of Melbourne, Melbourne, VIC (2020).

[55] B. Wimborne, L. R. Marotte, R. F. Mark, “The brain of the tammar wallaby (*Macropus eugenii*) in stereotaxic coordinates” (Australian National University, 2008).

[56] S. L. Cloherty, N. J. Hughes, M. A. Hietanen, P. S. Bhagavatula, G. J. Goodhill, M. R. Ibbotson, Sensory experience modifies feature map relationships in visual cortex. eLife 5, e13911 (2016). 27310531 10.7554/eLife.13911 PMC4911216

[57] I. Schießl, M. Stetter, J. E. Mayhew, N. McLoughlin, J. S. Lund, K. Obermayer, Blind signal separation from optical imaging recordings with extended spatial decorrelation. IEEE Trans. Biomed. Eng. 47, 573–577 (2000). 10851799 10.1109/10.841327

[58] M. A. Carreira-Perpinán, R. J. Lister, G. J. Goodhill, A computational model for the development of multiple maps in primary visual cortex. Cereb. Cortex 15, 1222–1233 (2005). 15616135 10.1093/cercor/bhi004

[59] M. Pachitariu, N. A. Steinmetz, S. N. Kadir, M. Carandini, K. D. Harris, Fast and accurate spike sorting of high-channel count probes with KiloSort, in *Proceedings of the Advances in Neural Information Processing Systems 29*, D. D. Lee, M. Sugiyama, U. V. Luxburg, I. Guyon, R. Garnett, Eds. (NIPS Proceedings, 2016).

[60] K. A. Ludwig, R. M. Miriani, N. B. Langhals, M. D. Joseph, D. J. Anderson, D. R. Kipke, Using a common average reference to improve cortical neuron recordings from microelectrode arrays. J. Neurophysiol. 101, 1679–1689 (2009). 19109453 10.1152/jn.90989.2008 PMC2666412

[61] C. Rossant, S. N. Kadir, D. F. M. Goodman, J. Schulman, M. L. D. Hunter, A. B. Saleem, A. Grosmark, M. Belluscio, G. H. Denfield, A. S. Ecker, A. S. Tolias, S. Solomon, G. Buzsáki, M. Carandini, K. D. Harris, Spike sorting for large, dense electrode arrays. Nat. Neurosci. 19, 634–641 (2016). 26974951 10.1038/nn.4268 PMC4817237

[62] A. Gharat, C. L. Baker Jr., Nonlinear Y-like receptive fields in the early visual cortex: An intermediate stage for building cue-invariant receptive fields from subcortical Y cells. J. Neurosci. 37, 998–1013 (2017). 28123031 10.1523/JNEUROSCI.2120-16.2016 PMC6597016

[63] V. Perry, A. Cowey, Retinal ganglion cells that project to the superior colliculus and pretectum in the macaque monkey. Neuroscience 12, 1125–1137 (1984). 6483194 10.1016/0306-4522(84)90007-1

[64] M. E. Garrett, I. Nauhaus, J. H. Marshel, E. M. Callaway, Topography and areal organization of mouse visual cortex. J. Neurosci. 34, 12587–12600 (2014). 25209296 10.1523/JNEUROSCI.1124-14.2014 PMC4160785

[65] S. G. Espinoza, H. C. Thomas, Retinotopic organization of striate and extrastriate visual cortex in the hooded rat. Brain Res. 272, 137–144 (1983). 6616189 10.1016/0006-8993(83)90370-0

[66] A. Hughes, Topographical relationships between the anatomy and physiology of the rabbit visual system. Doc. Ophthalmol. 30, 33–159 (1971). 5000058 10.1007/BF00142518

[67] M. I. Law, K. R. Zahs, M. P. Stryker, Organization of primary visual cortex (area 17) in the ferret. J. Comp. Neurol. 278, 157–180 (1988). 3068264 10.1002/cne.902780202

[68] M. Sesma, V. Casagrande, J. Kaas, Cortical connections of area 17 in tree shrews. J. Comp. Neurol. 230, 337–351 (1984). 6520238 10.1002/cne.902300303

[69] R. Tusa, L. Palmer, A. Rosenquist, The retinotopic organization of area 17 (striate cortex) in the cat. J. Comp. Neurol. 177, 213–235 (1978). 413845 10.1002/cne.901770204

[70] D. L. Adams, L. C. Sincich, J. C. Horton, Complete pattern of ocular dominance columns in human primary visual cortex. J. Neurosci. 27, 10391–10403 (2007). 17898211 10.1523/JNEUROSCI.2923-07.2007 PMC6673158

[71] A. Hughes, A schematic eye for the rat. Vision Res. 19, 569–588 (1979). 483586 10.1016/0042-6989(79)90143-3

[72] Z. Henderson, B. Finlay, K. Wikler, Development of ganglion cell topography in ferret retina. J. Neurosci. 8, 1194–1205 (1988). 3357016 10.1523/JNEUROSCI.08-04-01194.1988 PMC6569276

[73] R. Engelmann, L. Peichl, Unique distribution of somatostatin-immunoreactive cells in the retina of the tree shrew (*Tupaia belangeri*). Eur. J. Neurosci. 8, 220–228 (1996). 8713466 10.1111/j.1460-9568.1996.tb01183.x

[74] A. Hughes, A quantitative analysis of the cat retinal ganglion cell topography. J. Comp. Neurol. 163, 107–128 (1975). 1159109 10.1002/cne.901630107

[75] B. M. Wimborne, R. F. Mark, M. R. Ibbotson, Distribution of retinogeniculate cells in the tammar wallaby in relation to decussation at the optic chiasm. J. Comp. Neurol. 405, 128–140 (1999). 10022200

[76] X. Kong, K. Wang, X. Sun, R. E. Witt, Comparative study of the retinal vessel anatomy of rhesus monkeys and humans. Clin. Exp. Ophthalmol. 38, 629–634 (2010). 20584020 10.1111/j.1442-9071.2010.02290.x

[77] L. Silveira, C. Picanço-Diniz, E. Oswaldo-Cruz, Distribution and size of ganglion cells in the retinae of large Amazon rodents. Vis. Neurosci. 2, 221–235 (1989). 2562148 10.1017/s0952523800001140

[78] O. Dkhissi-Benyahya, A. Szel, W. J. Degrip, H. M. Cooper, Short and mid-wavelength cone distribution in a nocturnal strepsirrhine primate (*Microcebus murinus*). J. Comp. Neurol. 438, 490–504 (2001). 11559903 10.1002/cne.1330

[79] C. F. Ross, R. F. Kay, *Anthropoid Origins: New Visions* (Springer Science & Business Media, 2012).

[80] S. Robinson, G. Horsburgh, B. Dreher, M. McCall, Changes in the numbers of retinal ganglion cells and optic nerve axons in the developing albino rabbit. Dev. Brain Res. 35, 161–174 (1987). 10.1016/0165-3806(87)90041-1 3676835

[81] P. Johnson, S. Geller, B. Reese, Distribution, size and number of axons in the optic pathway of ground squirrels. Exp. Brain Res. 118, 93–104 (1998). 9547081 10.1007/s002210050258

[82] B. C. Samuels, J. T. Siegwart, W. Zhan, L. Hethcox, M. Chimento, R. Whitley, J. C. Downs, C. A. Girkin, A novel tree shrew (*Tupaia belangeri*) model of glaucoma. Invest. Ophthalmol. Vis. Sci. 59, 3136–3143 (2018). 30025140 10.1167/iovs.18-24261 PMC6018453

[83] K. O. Long, S. K. Fisher, The distributions of photoreceptors and ganglion cells in the California ground squirrel, *Spermophilus beecheyi*. J. Comp. Neurol. 221, 329–340 (1983). 6655087 10.1002/cne.902210308

[84] E. J. Debruyn III, “The organization and central terminations of retinal ganglion cells in the tree shrew (*Tupaia glis*),” thesis, Vanderbilt University, Nashville, TN (1983).

[85] K. Obermayer, G. G. Blasdel, Singularities in primate orientation maps. Neural Comput. 9, 555–575 (1997). 9097474 10.1162/neco.1997.9.3.555

[86] F. Wolf, T. Geisel, Spontaneous pinwheel annihilation during visual development. Nature 395, 73–78 (1998). 9738500 10.1038/25736

