# Supplementary material for: Orientation pinwheels in primary visual cortex of a highly visual marsupial
Source: Sci Adv. 2022 Sep 30;8(39):eabn0954. doi: 10.1126/sciadv.abn0954 (PMC9524828; doi:10.1126/sciadv.abn0954)
Supplement: Supplementary file 1 — Figs. S1 to S6 Tables S1 and S2 References [file sciadv.abn0954_sm.pdf]

Science Advances
AAAS

Supplementary Materials for

Orientation pinwheels in primary visual cortex of a highly visual marsupial

Young Jun Jung *et al.*

Corresponding author: Young Jun Jung, yjjung@aco.org.au

Sci. Adv. **8**, eabn0954 (2022)
DOI: 10.1126/sciadv.abn0954

This PDF file includes:

Figs. S1 to S6
Tables S1 and S2
References

The stereotaxic coordinates of Area 17 in the wallaby brain.

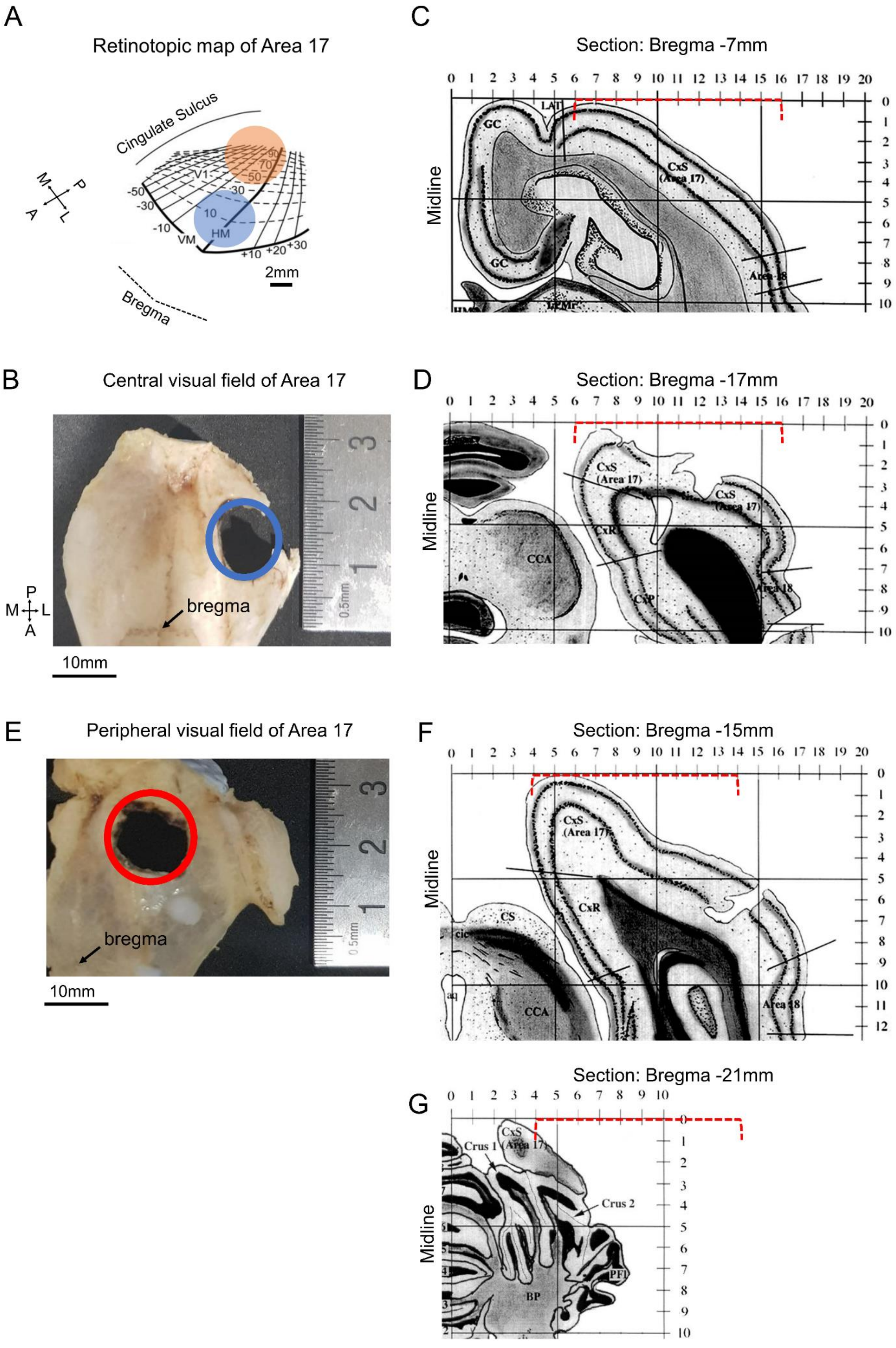

Figure S1. The stereotaxic coordinates of Area 17 in the wallaby brain. (A) A schematic diagram of the retinotopic map of wallaby area 17 adapted from *(25, 63)*. The circles represent the estimates of the ROI where the frontal and peripheral OP maps were taken. Blue circle = frontal visual field representation, Red circle = peripheral visual field representation. **(B, E)** Dorsal view of the skull of the Tammar wallaby showing Bregma and the craniotomy for frontal and peripheral recordings. **(C, D)** Sagittal sections of the wallaby brain from -7mm and -17mm posterior to Bregma for the frontal field craniotomy window, and **(F, G)** -15mm and -21mm posterior to Bregma for the peripheral field craniotomy window. Note the atlas only showed up to -22mm posterior to Bregma. The red bracket marks the location of the brain exposed by our craniotomy (Sourced from open access files: https://openresearch-repository.anu.edu.au/handle/1885/10264)*(55)*.

Mapping signals from green light imaging

Figure S2 (B,C) shows the differential maps generated by subtracting the responses from orthogonal stimuli., without ESD or the low-pass Gaussian filtering. As shown in Fig. S2 (A), the relative change in reflectance from the green light recording is strong relative to the noise. The blood vessel artefacts in the maps derived from green light images were removed using extended spatial decorrelation (ESD), as described above. This technique allowed for robust separation of the stimulus-specific intrinsic signals from the biological noise and vascular artefacts. There is a high degree of similarity between the OP map derived using ESD and that derived using the conventional method. The pinweel density calculated from the map produced with the ESD method was 2.93, with column spacing of 1.43mm and the pinwheel density calculated from the differential map was 2.99, with column spacing of 1.33mm. So, while ESD employs a more sophisticated analysis technique to separate stimulus-specific intrinsic signals from the biological noise and artefacts, the method does not produce maps that are considerably different to conventional methods for analysis of optical images.

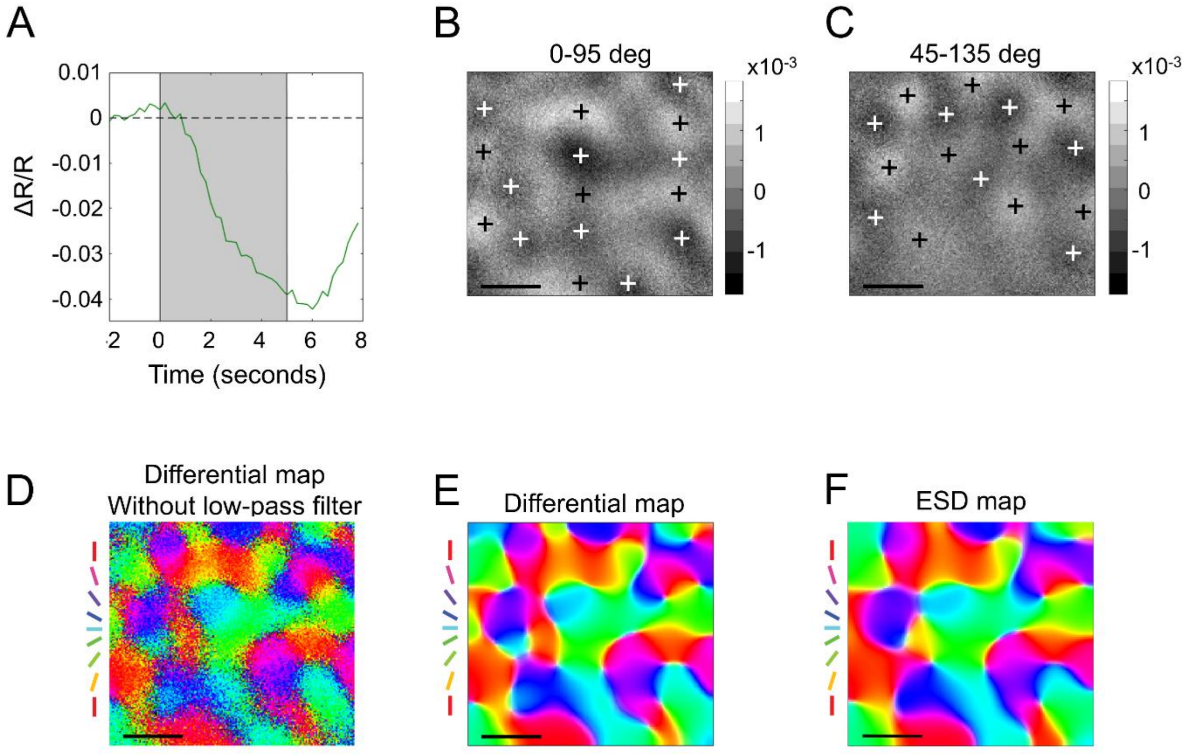

Figure S2. Optical imaging using green light (A) Time course of the relative change in reflectance ($\Delta R/R$) during a trial, averaged over all pixels and all trials, measured with green (520 nm) light. The shaded region shows the stimulus period. Differential maps generated by subtracting the responses from orthogonal stimuli. **(B)** 0-90deg and **(C)** 45-135deg maps without ESD or low-pass filtering. **(D)** OP map generated from differential imaging without low-pass filtering. **(E)** OP map generated using the conventional approach, i.e. differential imaging where orthogonal stimuli are subtracted from each other. **(F)** OP maps generated from the ESD. While ESD removes any biological noise and artefacts from the map, the spatial patterns of the two maps derived using the two different techniques are similar. Scale bar = 1mm.

We also tested the effect of low frequency noise on the pinwheel density by varying the high-pass cutoff wavelength (λ_{hp}) underlying the analysis. As a representative example, we present one wallaby map from the frontal representation. Fig. S3 shows the wallaby average pinwheel density for various cutoff wavelengths (λ_{hp}). For each value of (λ_{hp}), the pinwheel density (ρ) was calculated. Apart from the high-pass cutoff wavelength λ_{hp}, all parameters were identical for each data point. We found that the average pinwheel densities were similar between λhp = 100 and λhp = 700, which highlighs the robustness of the pinwheel density estimation over a broad range of cut-off wavelengths.

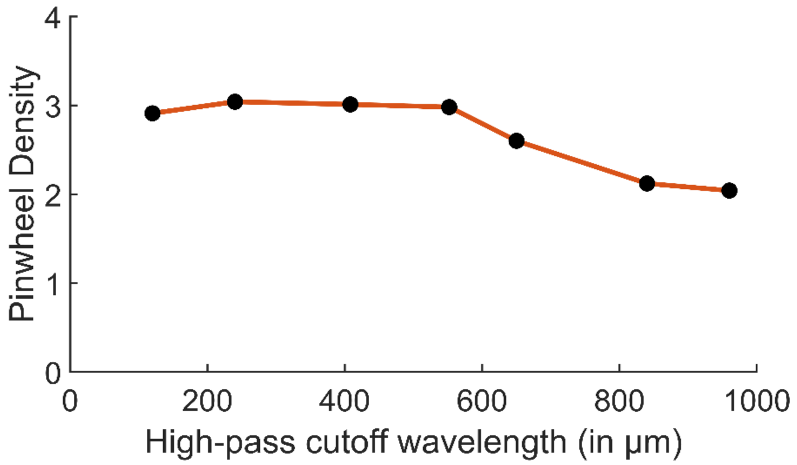

Figure S3. Testing the robustness of pinwheel density estimation against variations in the high-pass filtering. Pinwheel density in one image from a frontal representation for various high-pass filters. Values from left to right = 2.91, 3.04, 3.01, 2.98, 2.60, 2.12, 2.04. The pinwheel density was not greatly changed between λhp = 100 and λhp = 700.

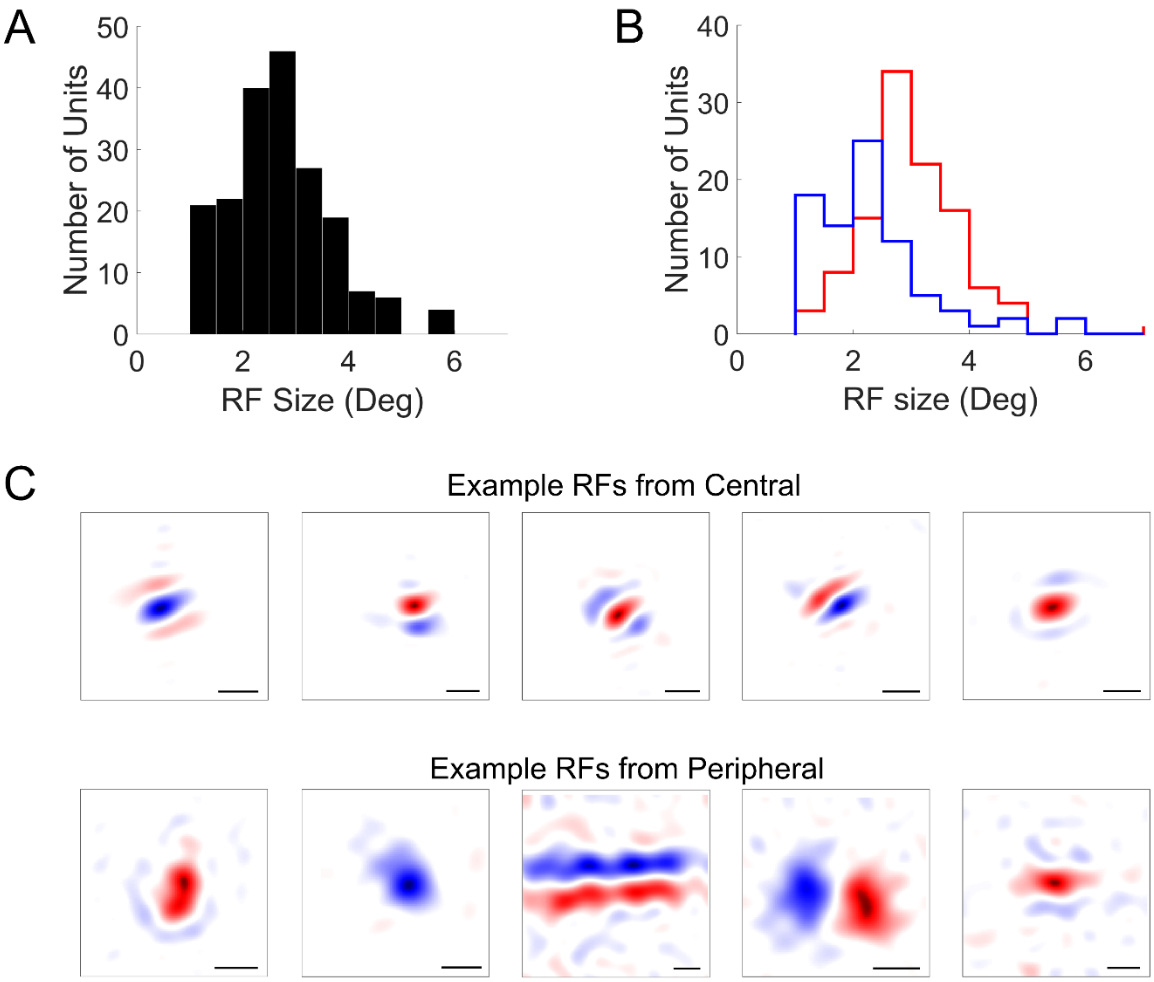

Figure S4. The distribution of Receptive Field sizes (in degrees) across 195 single unit (SU) recordings in V1. A) Histogram shows the distribution of RF sizes across all 195 SUs recorded in V1. **B)** Histogram shows the distribution of RF Sizes along the longest axis from the SUs recorded in the central visual field (blue) and in the peripheral visual field (red). We found that the mean and the distribution was significantly different (Central: 2.3±0.97 deg, mean ± std; peripheral: 3.1±0.97 deg, mean ± std, $p<0.05$). **C)** Example RFs from single units recorded in the central visual field (n=83) and D) in the peripheral visual field (n=112). Black scale bar indicates 1° of visual field

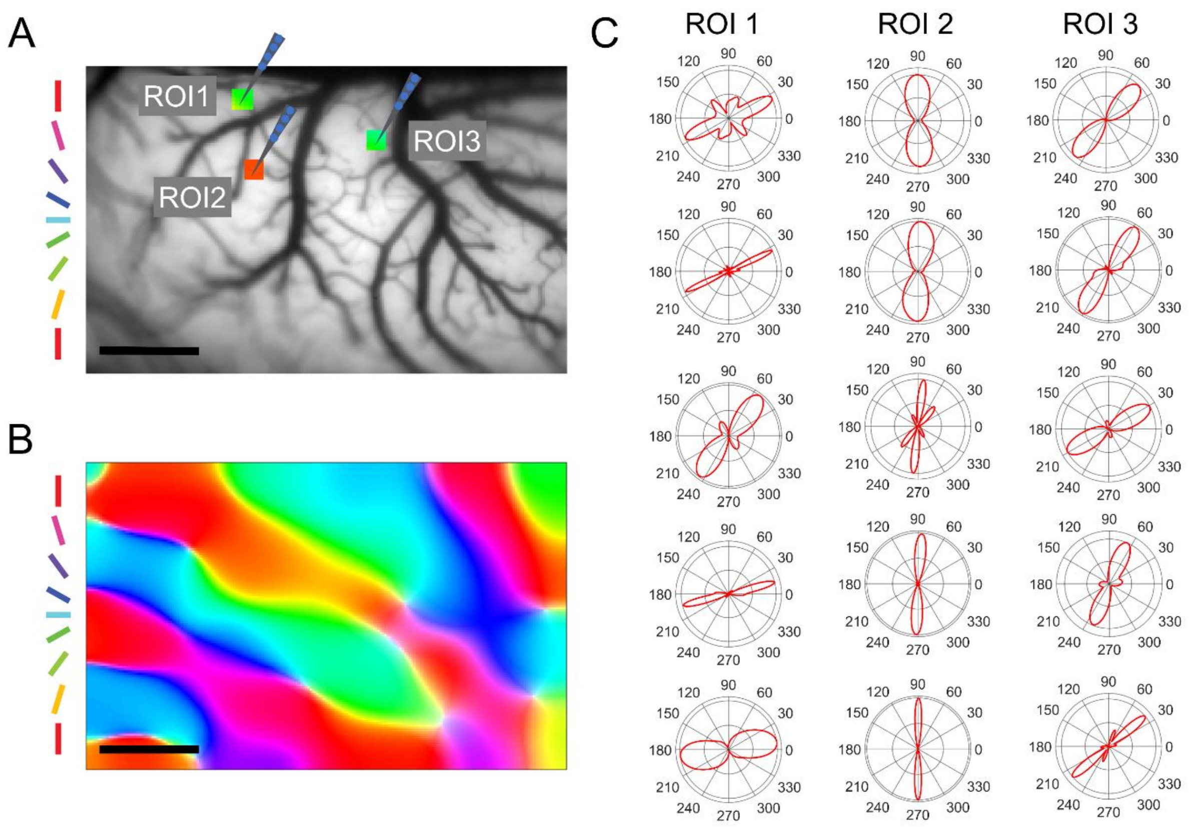

Figure S5. Single unit recordings from the superficial layers of the cortex (200-1000 µm) were obtained. A) We show the cortical surface imaged under green light with square symbols overlaid indicating the locations of electrode penetrations. Black bar = 1mm. We inserted electrodes into different orientation columns in Area 17 to compare measures of orientation preferences from single-unit recordings with the corresponding measures from the OP maps. 10 x 10 pixel ROIs were placed around each of the electrode tracks. The three coloured boxes represent the vector average of the pixels within the ROIs. ROI 1 = 34°, ROI 2 = 88°, ROI 3 = 22°. **B)** Colour-coded orientation preference map showing the preferred orientation for every region of interest. Orientation preference is colour-coded according to the scheme in the legend. Scale bars = 1mm. C) Example polar plots of the single unit recordings from the three ROIs. ROI 1 = 30°±17°, ROI 2 = 89°±13°, ROI 3 = 49° ±28°.

Orientation map in Animal 1.

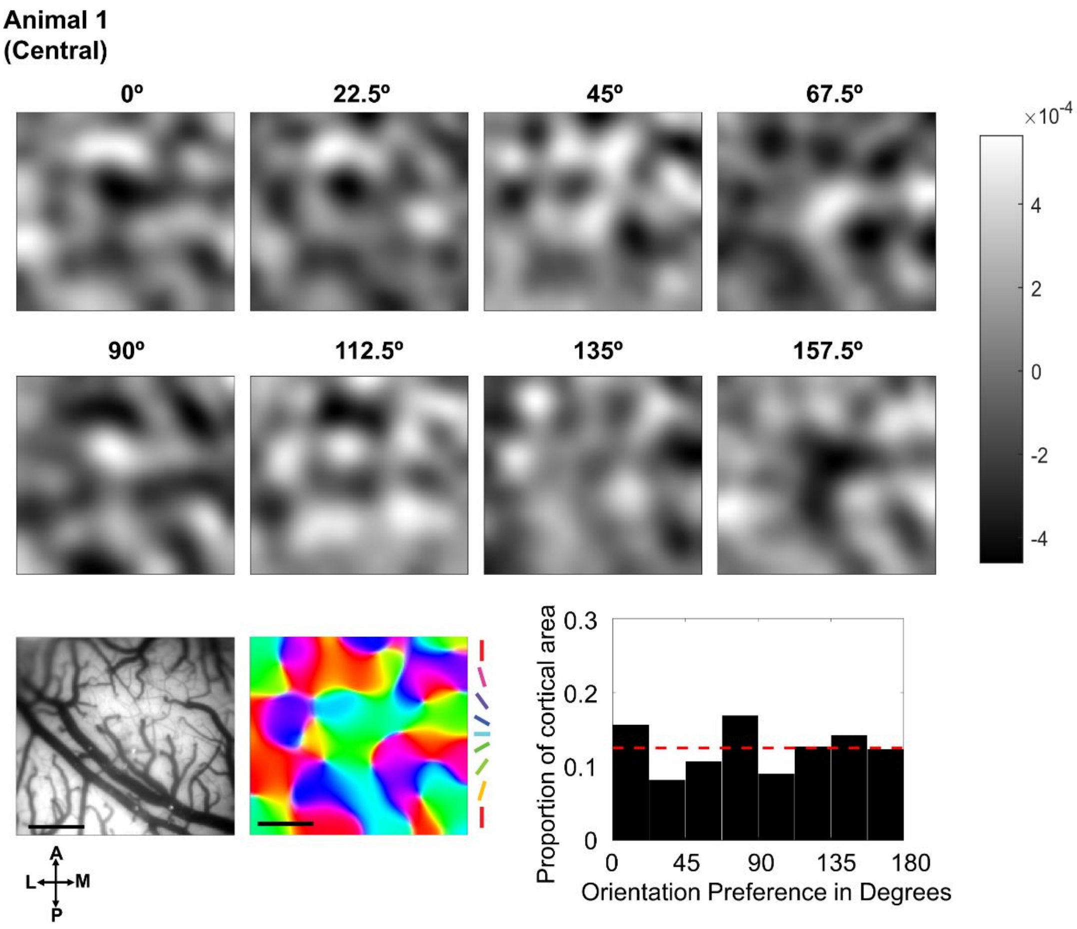

Orientation map in Animal 2.

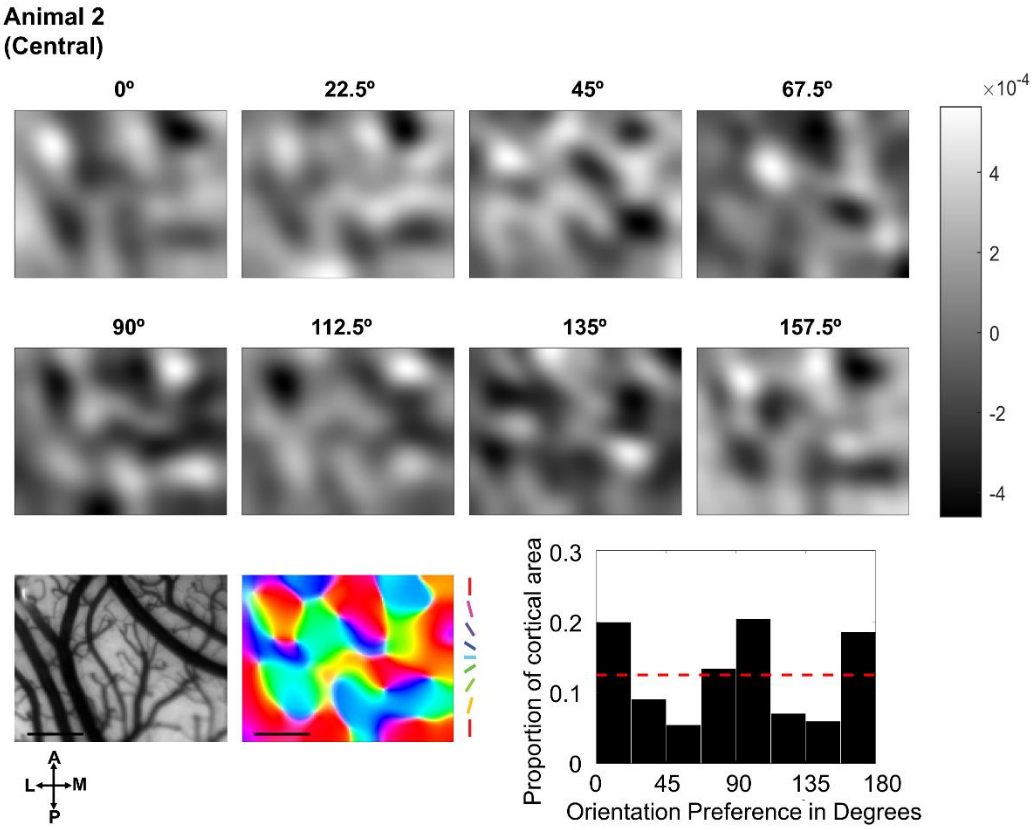

Orientation map in Animal 3.

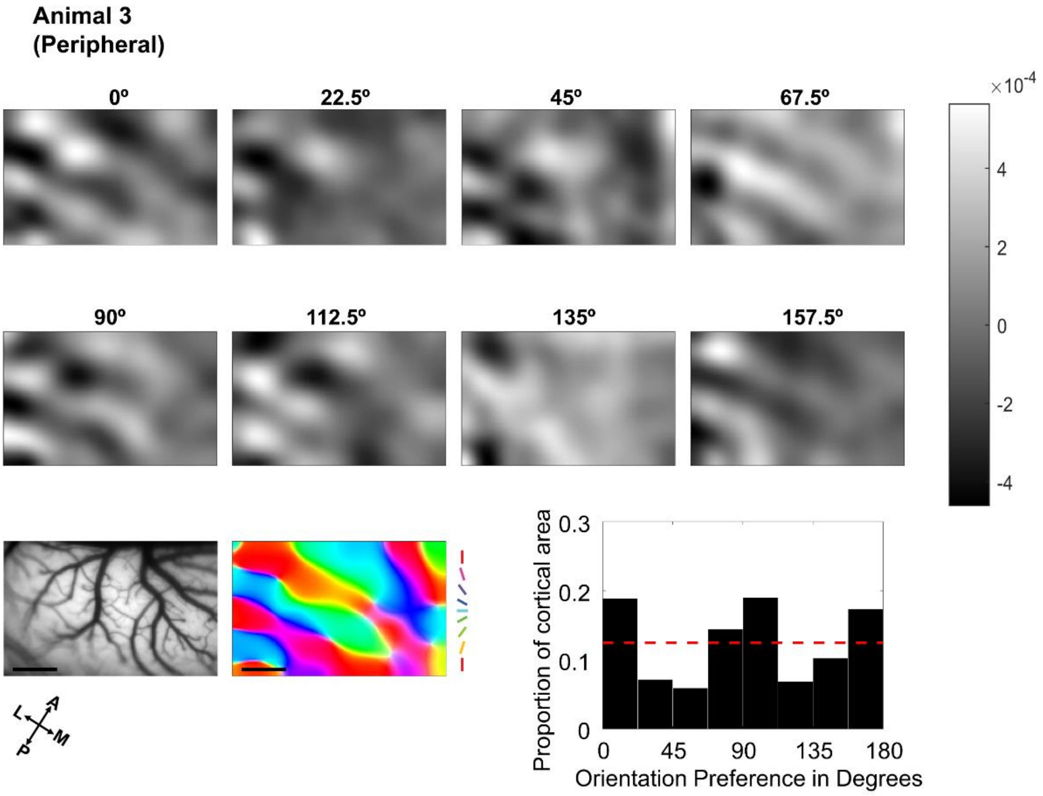

Orientation map in Animal 4.

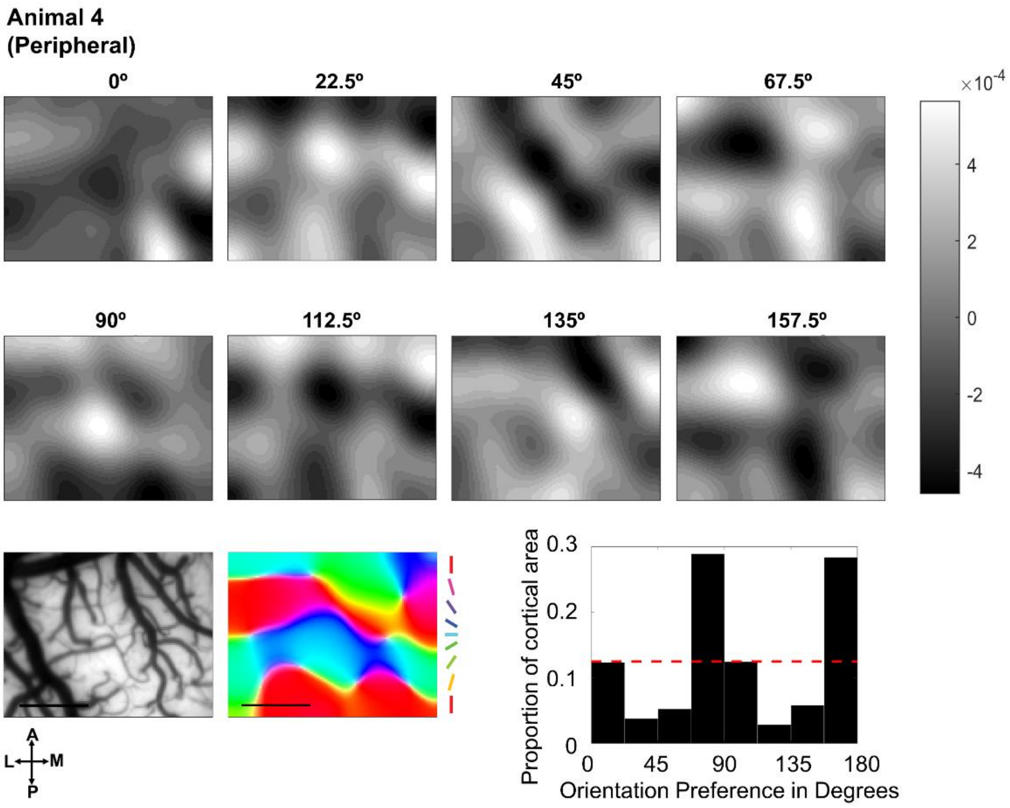

Orientation map in Animal 5.

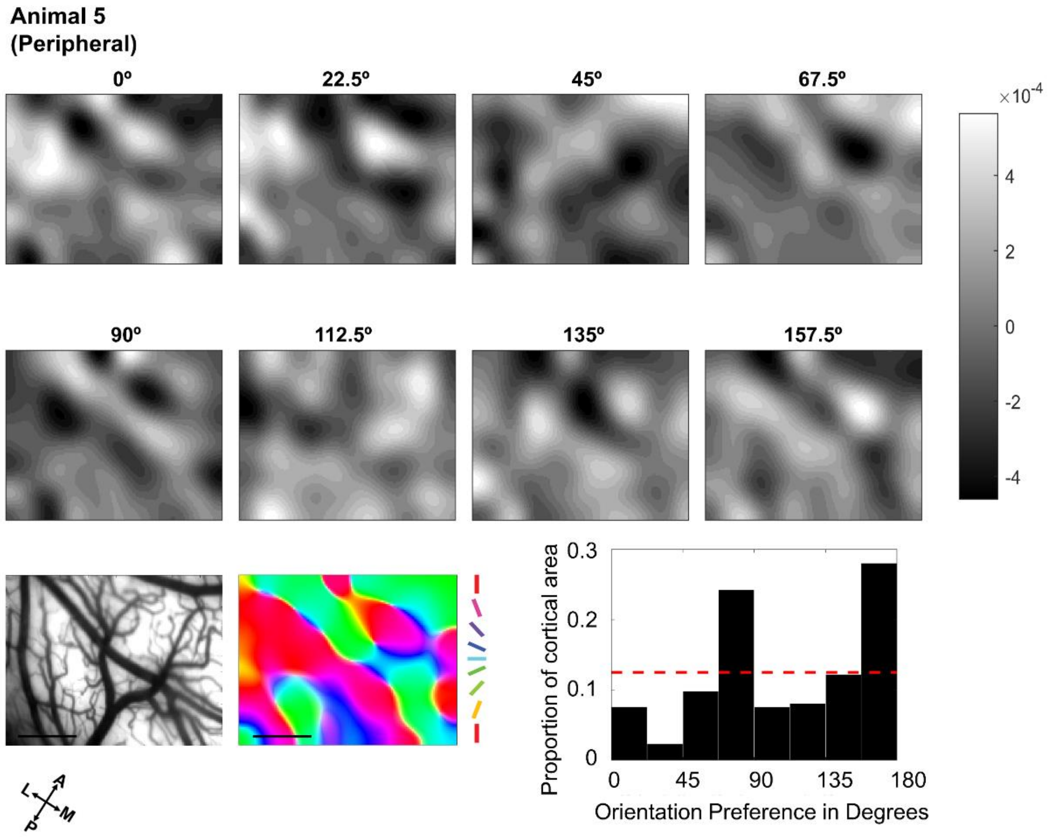

Orientation map in Animal 6.

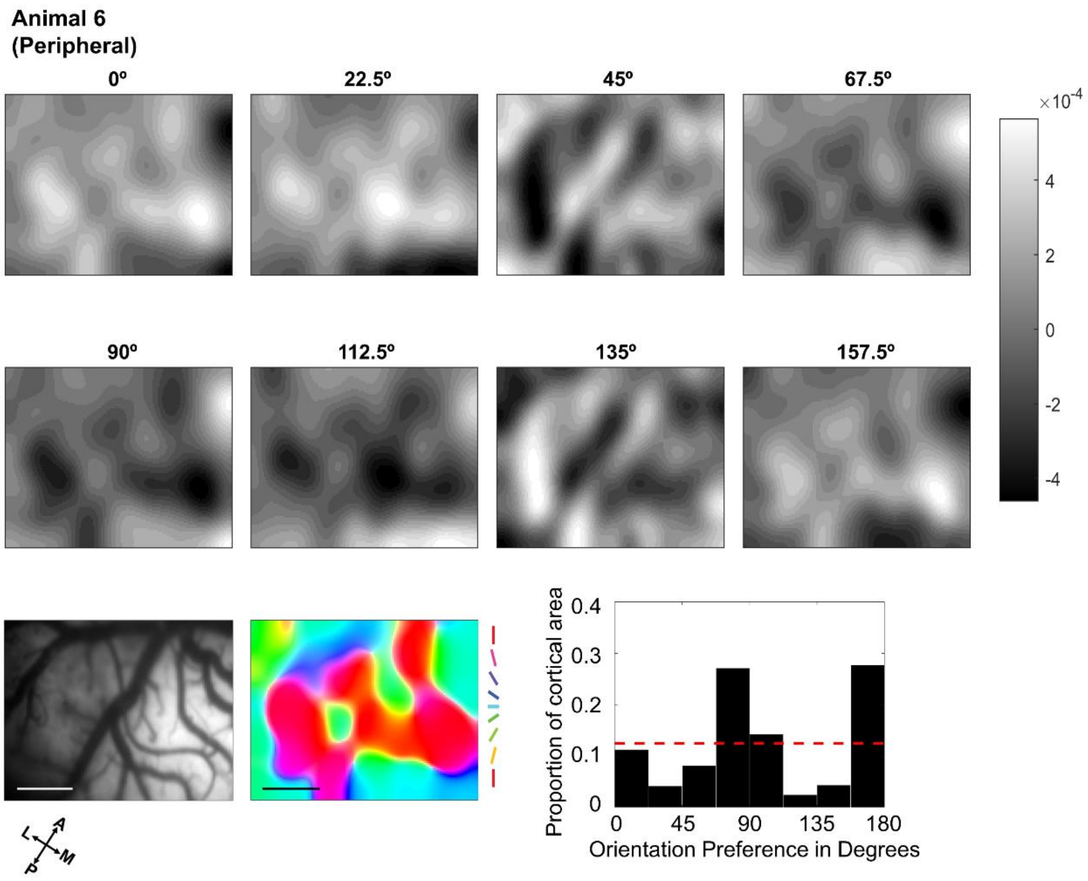

Figure S6. Orientation maps from Animals 1-6. For each animal, eight single condition maps in response to moving gratings are presented. The greyscale represents the response magnitude (dark areas represent high activity). (Lower left) The image of the cortical surface illuminated with green light. A, Anterior; P, posterior; M, medial; L, lateral. (Lower middle) Colour-coded orientation preference map showing the preferred orientation for every region of interest. Orientation preference is colour-coded according to the scheme in the legend. Scale bars = 1mm. (Lower right) Proportion of cortical area representing different orientations from right eye stimulation. The red dashed line at a frequency of 1/8 represents uniform distribution.

Table S1. Experimental Data on retinal and V1 cell density.

	Mouse	Rat	Rabbit	Squirrel	Ferret	TS*	Cat	Wallaby	Macaque	Agouti	M.lemur
**V1 Size (mm^2)**	3 *(64)*	7 *(65)*	80 *(66)*	82 *(12)*	83 *(67)*	83 *(20,68)*	380 *(69)*	135 *(25)*	1257 *(70)*	320 *(19,13)*	48 *(18)*
**Retina Size (mm^2)**	15 *(46)*	52 *(71)*	436 *(42)*	205 *(42)*	84 *(72)*	122 *(73)*	510 *(74)*	432 *(75)*	636 *(76)*	536 *(77)*	130 *(78)*
**Size Ratio (V1/Retina)**	0.20	0.13	0.18	0.40	0.98	0.60	0.75	0.32	1.98	0.60	0.36
**2D Number of V1 neurons ($x10^3$)$^+$**	7 *(51)*	14 *(51)*	162 *(51)*	166 *(51)*	169 *(51)*	149 *(51)*	681 *(51)*	273 *(51)*	3812 *(51)*	-	-
**Number of RGC ($x10^3$)**	70 *(79)*	110 *(79)*	550 *(80)*	1200 *(81)*	90 *(72)*	305 *(82)*	247 *(74)*	360 *(75)*	1600 *(79)*	482 *(77)*	-
**Cell number ratio, V1/RGC)**	0.09	0.13	0.29	0.14	1.87	0.49	2.76	0.76	2.38	-	-
**Centro-peripheral density (CP ratio)**	2.7 *(46,17)*	1.4 *(71, 17)*	2.7 *(42,17)*	3.2 *(83,17)*	11.0 *(72,17)*	7.6 *(84,17)*	36.4 *(74,17)*	20 *(75,7)*	60.0 *(63,17)*	2.9 *(77,17)*	22 *(78,17)*

*TS = Tree Shrew

+ 2D number of V1 neurons were calculated by Jang et al. *(51).*

Table S2: Pinwheel density (ρ) and column spacing (Λ) across various species

Species	**Bodyweight (g)**	**Average pinwheel Density (ρ)**	**Column Spacing (Λ)**
Mouse	25g *(12)*	0	0
Mouse lemurs	60g *(18)*	3.15 *(18)*	0.54 *(18)*
Galago	150g *(9)*	3.15 *(9)*	0.49 *(9)*
Tree shrew	200g *(12)*	3.12 *(9)*	0.47 *(9)*
Ferret	800g *(12)*	3.15 *(9)*	0.51 *(9)*
Squirrel monkey	1100g *(9)*	2.90 (*85, 86*)	0.51 *(85, 86)*
Cat	3250g *(12)*	3.09 *(52)*	1.10 *(36, 71, 86)*
Wallaby	4800g (own data)	2.62 (own data)	1.43 (own data)
Macaque	8000g *(12)*	3.75 (*70, 86*)	0.68 (*70, 86*)

REFERENCES AND NOTES

1. J. Kremkow, J. Jin, Y. Wang, J. M. Alonso, Principles underlying sensory map topography in primary visual cortex. *Nature* **533**, 52–57 (2016).

2. J. Cang, R. C. Rentería, M. Kaneko, X. Liu, D. R. Copenhagen, M. P. Stryker, Development of precise maps in visual cortex requires patterned spontaneous activity in the retina. *Neuron* **48**, 797–809 (2005).

3. F. Briggs. Organizing principles of cortical layer 6. *Front. Neural Circuits* **4**, 3 (2010).

4. T. Bonhoeffer, A. Grinvald, Iso-orientation domains in cat visual cortex are arranged in pinwheel-like patterns. *Nature* **353**, 429–431 (1991).

5. B. Chapman, M. P. Stryker, T. Bonhoeffer, Development of orientation preference maps in ferret primary visual cortex. *J. Neurosci.* **16**, 6443–6453 (1996).

6. W. H. Bosking, Y. Zhang, B. Schofield, D. Fitzpatrick, Orientation selectivity and the arrangement of horizontal connections in tree shrew striate cortex. *J. Neurosci.* **17**, 2112–2127 (1997).

7. D. Hubel, T. Wiesel, Anatomical demonstration of columns in the monkey striate cortex. *Nature* **221**, 747–750 (1969).

8. G. G. Blasdel, Orientation selectivity, preference, and continuity in monkey striate cortex. *J. Neurosci.* **12**, 3139–3161 (1992).

9. M. Kaschube, M. Schnabel, S. Löwel, D. M. Coppola, L. E. White, F. Wolf, Universality in the evolution of orientation columns in the visual cortex. *Science* **330**, 1113–1116 (2010).

10. D. B. Chklovskii, A. A. Koulakov, Maps in the brain: What can we learn from them?. *Annu. Rev. Neurosci.* **27**, 369–392 (2004).

11. K. Ohki, S. Chung, Y. H. Ch'ng, P. Kara, R. C. Reid, Functional imaging with cellular resolution reveals precise micro-architecture in visual cortex. *Nature* **433**, 597–603 (2005).

12. S. D. Van Hooser, J. A. F. Heimel, S. Chung, S. B. Nelson, L. J. Toth, Orientation selectivity without orientation maps in visual cortex of a highly visual mammal. *J. Neurosci.* **25**, 19–28 (2005).

13. D. N. Ferreiro, S. A. Conde-Ocazionez, J. H. N. Patriota, L. C. Souza, M. F. Oliveira, F. Wolf, K. E. Schmidt, Spatial clustering of orientation preference in primary visual cortex of the large rodent agouti. *Iscience* **24**, 101882 (2021).

14. D. L. Ringach, P. J. Mineault, E. Tring, N. D. Olivas, P. Garcia-Junco-Clemente, J. T. Trachtenberg, Spatial clustering of tuning in mouse primary visual cortex. *Nat. Commun.* **7**, 1–9 (2016).

15. S. Kondo, T. Yoshida, K. Ohki, Mixed functional microarchitectures for orientation selectivity in the mouse primary visual cortex. *Nat. Commun.* **7**, 13210 (2016).

16. A. Hughes, in *The Visual System in Vertebrates* (Springer, 1977), pp. 613–756.

17. M. Ibbotson, Y. J. Jung, Origins of functional organization in the visual cortex. *Front. Syst. Neurosci.* **14**, 10 (2020).

18. C. L. A. Ho, R. Zimmermann, J. D. Flórez Weidinger, M. Prsa, M. Schottdorf, S. Merlin, T. Okamoto, K. Ikezoe, F. Pifferi, F. Aujard, A. Angelucci, F. Wolf, D. Huber, Orientation preference maps in *Microcebus murinus* reveal size-invariant design principles in primate visual cortex. *Curr. Biol.* **31**, 733–741..e7 (2021).

19. K. E. Schmidt, F. Wolf, Punctuated evolution of visual cortical circuits? Evidence from the large rodent *Dasyprocta leporina*, and the tiny primate *Microcebus murinus*. *Curr. Opin. Neurobiol.* **71**, 110–118 (2021).

20. W. Keil, M. Kaschube, M. Schnabel, Z. F. Kisvarday, S. Löwel, D. M. Coppola, L. E. White, F. Wolf, Response to comment on “universality in the evolution of orientation columns in the visual cortex”. *Science* **336**, 413–413 (2012).

21. M. Kaschube, Neural maps versus salt-and-pepper organization in visual cortex. *Curr. Opin. Neurobiol.* **24**, 95–102 (2014).

22. Z.-X. Luo, C.-X. Yuan, Q.-J. Meng, Q. Ji, A Jurassic eutherian mammal and divergence of marsupials and placentals. *Nature* **476**, 442–445 (2011).

23. K. W. Ashwell, Anterior commissure versus corpus callosum: A quantitative comparison across mammals. *Fortschr. Zool.* **119**, 126–136 (2016).

24. A. Grinvald, E. Lieke, R. D. Frostig, C. D. Gilbert, T. N. Wiesel, Functional architecture of cortex revealed by optical imaging of intrinsic signals. *Nature* **324**, 361–364 (1986).

25. T. Vidyasagar, J. Wye-Dvorak, G. Henry, R. Mark, Cytoarchitecture and visual field representation in area 17 of the tammar wallaby (*Macropus eugenii*). *J Comp Neurol* **325**, 291–300 (1992).

26. M. Ibbotson, R. Mark, Orientation and spatiotemporal tuning of cells in the primary visual cortex of an Australian marsupial, the wallaby *Macropus eugenii*. *J. Comp. Physiol. A* **189**, 115–123 (2003).

27. M. Weliky, W. H. Bosking, D. Fitzpatrick, A systematic map of direction preference in primary visual cortex. *Nature* **379**, 725–728 (1996).

28. C. Rocha-Miranda, R. Linden, E. Volchan, R. Lent, R. Bombardieri Jr, Receptive field properties of single units in the opossum striate cortex. *Brain Res.* **104**, 197–219 (1976).

29. J. C. Dooley, M. S. Donaldson, L. A. Krubitzer, Cortical plasticity following stripe rearing in the marsupial *Monodelphis domestica*: Neural response properties of V1. *J. Neurophysiol.* **117**, 566–581 (2017).

30. D. P. Crewther, S. G. Crewther, K. J. Sanderson, Primary visual cortex in the brushtailed possum: Receptive field properties and corticocortical connections. *Brain Behav. Evol.* **24**, 184–197 (1984).

31. D. H. Hubel, T. N. Wiesel, Receptive fields, binocular interaction and functional architecture in the cat’s visual cortex. *J. Physiol.* **160**, 106–154 (1962).

32. D. H. Hubel, T. N. Wiesel, Receptive fields and functional architecture of monkey striate cortex. *J. Physiol.* **195**, 215–243 (1968).

33. A. Almasi, H. Meffin, S. L. Cloherty, Y. Wong, M. Yunzab, M. R. Ibbotson, Mechanisms of feature selectivity and invariance in primary visual cortex. *Cereb. Cortex* **30**, 5067–5087 (2020).

34. J. Stone, B. Dreher, A. Leventhal, Hierarchical and parallel mechanisms in the organization of visual cortex. *Brain Res. Rev.* **1**, 345–394 (1979).

35. S. D. Van Hooser, Similarity and diversity in visual cortex: Is there a unifying theory of cortical computation? *Neuroscientist* **13**, 639–656 (2007).

36. S. Chenchal Rao, L. J. Toth, M. Sur, Optically imaged maps of orientation preference in primary visual cortex of cats and ferrets. *J Comp Neurol* **387**, 358–370 (1997).

37. P. Clarke, I. Donaldson, D. Whitteridge, Binocular visual mechanisms in cortical areas I and II of the sheep. *J. Physiol.* **256**, 509–526 (1976).

38. Y. E. Zhang, P. Landback, M. D. Vibranovski, M. Long, Accelerated recruitment of new brain development genes into the human genome. *PLOS Biol.* **9**, e1001179 (2011).

39. D. L. Silver, Genomic divergence and brain evolution: How regulatory DNA influences development of the cerebral cortex. *Bioessays* **38**, 162–171 (2016).

40. S. Collin, in *Adaptive Mechanisms in the Ecology of Vision* (Springer, 1999), pp. 509–535.

41. S. P. Collin, A web-based archive for topographic maps of retinal cell distribution in vertebrates. *Clin. Exp. Optom.* **91**, 85–95 (2008).

42. C. W. Oyster, E. S. Takahashi, D. C. Hurst, Density, soma size, and regional distribution of rabbit retinal ganglion cells. *J. Neurosci.* **1**, 1331–1346 (1981).

43. A. Shinozaki, Y. Hosaka, T. Imagawa, M. Uehara, Topography of ganglion cells and photoreceptors in the sheep retina. *J Comp Neurol* **518**, 2305–2315 (2010).

44. A. Navarro-Sempere, Y. Segovia, M. García, Comparative analysis of retinal ganglion cell topography and behavioral ecology in Australian marsupials. *Int. J. Morphol.* **36**, 248–257 (2018).

45. F. Knolle, R. P. Goncalves, A. J. Morton, Sheep recognize familiar and unfamiliar human faces from two-dimensional images. *R. Soc. Open Sci.* **4**, 171228 (2017).

46. U. Drager, J. Olsen, Ganglion-cell distribution in the retina of the mouse. *Invest. Ophthalmol. Vis. Sci.* **20**, 285–293 (1981).

47. P. McGreevy, T. D. Grassi, A. M. Harman, A strong correlation exists between the distribution of retinal ganglion cells and nose length in the dog. *Brain Behav. Evol.* **63**, 13–22 (2004).

48. J. Kremkow, J.-M. Alonso, Thalamocortical circuits and functional architecture. *Annu. Rev. Vision Sci.* **4**, 263–285 (2018).

49. C. F. Stevens, An evolutionary scaling law for the primate visual system and its basis in cortical function. *Nature* **411**, 193–195 (2001).

50. J. M. McFarland, Y. Cui, D. A. Butts, Inferring nonlinear neuronal computation based on physiologically plausible inputs. *PLOS Comput. Biol.* **9**, e1003143 (2013).

51. J. Jang, M. Song, S.-B. Paik, Retino-cortical mapping ratio predicts columnar and salt-and-pepper organization in mammalian visual cortex. *Cell Rep.* **30**, 3270–3279.e3 (2020).

52. M. Schottdorf, W. Keil, D. Coppola, L. E. White, F. Wolf, Random wiring, ganglion cell mosaics, and the functional architecture of the visual cortex. *PLOS Comput. Biol.* **11**, e1004602 (2015).

53. A. Shmuel, A. Grinvald, Functional organization for direction of motion and its relationship to orientation maps in cat area 18. *J. Neurosci.* **16**, 6945–6964 (1996).

54. Y. J. Jung, thesis, University of Melbourne, Melbourne, VIC (2020).

55. B. Wimborne, L. R. Marotte, R. F. Mark, "The brain of the tammar wallaby (*Macropus eugenii*) in stereotaxic coordinates" (Australian National University, 2008).

56. S. L. Cloherty, N. J. Hughes, M. A. Hietanen, P. S. Bhagavatula, G. J. Goodhill, M. R. Ibbotson, Sensory experience modifies feature map relationships in visual cortex. *eLife* **5**, e13911 (2016).

57. I. Schießl, M. Stetter, J. E. Mayhew, N. McLoughlin, J. S. Lund, K. Obermayer, Blind signal separation from optical imaging recordings with extended spatial decorrelation. *IEEE Trans. Biomed. Eng.* **47**, 573–577 (2000).

58. M. A. Carreira-Perpinán, R. J. Lister, G. J. Goodhill, A computational model for the development of multiple maps in primary visual cortex. *Cereb. Cortex* **15**, 1222–1233 (2005).

59. M. Pachitariu, N. A. Steinmetz, S. N. Kadir, M. Carandini, K. D. Harris, Fast and accurate spike sorting of high-channel count probes with KiloSort, in *Proceedings of the Advances in Neural Information Processing Systems 29*, D. D. Lee, M. Sugiyama, U. V. Luxburg, I. Guyon, R. Garnett, Eds. (NIPS Proceedings, 2016).

60. K. A. Ludwig, R. M. Miriani, N. B. Langhals, M. D. Joseph, D. J. Anderson, D. R. Kipke, Using a common average reference to improve cortical neuron recordings from microelectrode arrays. *J. Neurophysiol.* **101**, 1679–1689 (2009).

61. C. Rossant, S. N. Kadir, D. F. M. Goodman, J. Schulman, M. L. D. Hunter, A. B. Saleem, A. Grosmark, M. Belluscio, G. H. Denfield, A. S. Ecker, A. S. Tolias, S. Solomon, G. Buzsáki, M. Carandini, K. D. Harris, Spike sorting for large, dense electrode arrays. *Nat. Neurosci.* **19**, 634–641 (2016).

62. A. Gharat, C. L. Baker Jr, Nonlinear Y-like receptive fields in the early visual cortex: An intermediate stage for building cue-invariant receptive fields from subcortical Y cells. *J. Neurosci.* **37**, 998–1013 (2017).

63. V. Perry, A. Cowey, Retinal ganglion cells that project to the superior colliculus and pretectum in the macaque monkey. *Neuroscience* **12**, 1125–1137 (1984).

64. M. E. Garrett, I. Nauhaus, J. H. Marshel, E. M. Callaway, Topography and areal organization of mouse visual cortex. *J. Neurosci.* **34**, 12587–12600 (2014).

65. S. G. Espinoza, H. C. Thomas, Retinotopic organization of striate and extrastriate visual cortex in the hooded rat. *Brain Res.* **272**, 137–144 (1983).

66. A. Hughes, Topographical relationships between the anatomy and physiology of the rabbit visual system. *Doc. Ophthalmol.* **30**, 33–159 (1971).

67. M. I. Law, K. R. Zahs, M. P. Stryker, Organization of primary visual cortex (area 17) in the ferret. *J Comp Neurol* **278**, 157–180 (1988).

68. M. Sesma, V. Casagrande, J. Kaas, Cortical connections of area 17 in tree shrews. *J Comp Neurol* **230**, 337–351 (1984).

69. R. Tusa, L. Palmer, A. Rosenquist, The retinotopic organization of area 17 (striate cortex) in the cat. *J. Comp. Neurol.* **177**, 213–235 (1978).

70. D. L. Adams, L. C. Sincich, J. C. Horton, Complete pattern of ocular dominance columns in human primary visual cortex. *J. Neurosci.* **27**, 10391–10403 (2007).

71. A. Hughes, A schematic eye for the rat. *Vision Res.* **19**, 569–588 (1979).

72. Z. Henderson, B. Finlay, K. Wikler, Development of ganglion cell topography in ferret retina. *J. Neurosci.* **8**, 1194–1205 (1988).

73. R. Engelmann, L. Peichl, Unique distribution of somatostatin-immunoreactive cells in the retina of the tree shrew (*Tupaia belangeri*). *Eur. J. Neurosci.* **8**, 220–228 (1996).

74. A. Hughes, A quantitative analysis of the cat retinal ganglion cell topography. *J Comp Neurol* **163**, 107–128 (1975).

75. B. M. Wimborne, R. F. Mark, M. R. Ibbotson, Distribution of retinogeniculate cells in the tammar wallaby in relation to decussation at the optic chiasm. *J. Comp. Neurol.* **405**, 128–140 (1999).

76. X. Kong, K. Wang, X. Sun, R. E. Witt, Comparative study of the retinal vessel anatomy of rhesus monkeys and humans. *Clin. Exp. Ophthalmol.* **38**, 629–634 (2010).

77. L. Silveira, C. Picanço-Diniz, E. Oswaldo-Cruz, Distribution and size of ganglion cells in the retinae of large Amazon rodents. *Vis. Neurosci.* **2**, 221–235 (1989).

78. O. Dkhissi-Benyahya, A. Szel, W. J. Degrip, H. M. Cooper, Short and mid-wavelength cone distribution in a nocturnal strepsirrhine primate (*Microcebus murinus*). *J Comp Neurol* **438**, 490–504 (2001).

79. C. F. Ross, R. F. Kay, *Anthropoid Origins: New Visions* (Springer Science & Business Media, 2012).

80. S. Robinson, G. Horsburgh, B. Dreher, M. McCall, Changes in the numbers of retinal ganglion cells and optic nerve axons in the developing albino rabbit. *Dev. Brain Res.* **35**, 161–174 (1987).

81. P. Johnson, S. Geller, B. Reese, Distribution, size and number of axons in the optic pathway of ground squirrels. *Exp. Brain Res.* **118**, 93–104 (1998).

82. B. C. Samuels, J. T. Siegwart, W. Zhan, L. Hethcox, M. Chimento, R. Whitley, J. C. Downs, C. A. Girkin, A novel tree shrew (*Tupaia belangeri*) model of glaucoma. *Invest. Ophthalmol. Vis. Sci.* **59**, 3136–3143 (2018).

83. K. O. Long, S. K. Fisher, The distributions of photoreceptors and ganglion cells in the California ground squirrel, *Spermophilus beecheyi*. *J. Comp. Neurol.* **221**, 329–340 (1983).

84. E. J. Debruyn III, “The organization and central terminations of retinal ganglion cells in the tree shrew (*Tupaia glis*),” thesis, Vanderbilt University, Nashville, TN (1983).

85. K. Obermayer, G. G. Blasdel, Singularities in primate orientation maps. *Neural Comput.* **9**, 555–575 (1997).

86. F. Wolf, T. Geisel, Spontaneous pinwheel annihilation during visual development. *Nature* **395**, 73–78 (1998).
